# The Effectiveness of a ‘Train the Trainer’ Model of Resuscitation Education for Rural Peripheral Hospital Doctors in Sri Lanka 

**DOI:** 10.1371/journal.pone.0079491

**Published:** 2013-11-08

**Authors:** Bishan N. Rajapakse, Teresa Neeman, Andrew H. Dawson

**Affiliations:** 1 Australian National University, Canberra, Australia; 2 South Asian Clinical Toxicology Research Collaboration, University of Peradeniya, Peradeniya, Sri Lanka; 3 Statistical Consulting Unit, Australian National University, Canberra, Australia; 4 Central Clinical School, Royal Prince Alfred Hospital, University of Sydney, Sydney, Australia; San Raffaele Scientific Institute, Italy

## Abstract

**Background:**

Sri Lankan rural doctors based in isolated peripheral hospitals routinely resuscitate critically ill patients but have difficulty accessing training. We tested a train-the-trainer model that could be utilised in isolated rural hospitals.

**Methods:**

Eight selected rural hospital non-specialist doctors attended a 2-day instructor course. These “trained trainers” educated their colleagues in advanced cardiac life support at peripheral hospital workshops and we tested their students in resuscitation knowledge and skills pre and post training, and at 6- and 12-weeks. Knowledge was assessed through 30 multiple choice questions (MCQ), and resuscitation skills were assessed by performance in a video recorded simulated scenario of a cardiac arrest using a Resuci Anne Skill Trainer mannequin.

**Results/Discussion/Conclusion:**

Fifty seven doctors were trained. Pre and post training assessment was possible in 51 participants, and 6-week and 12-week follow up was possible for 43, and 38 participants respectively. Mean MCQ scores significantly improved over time (*p*<0.001), and a significant improvement was noted in “average ventilation volume”, “compression count”, and “compressions with no error”, “adequate depth”, “average depth”, and “compression rate” (*p*<0.01). The proportion of participants with compression depth ≥40mm increased post intervention (*p*<0.05) and at 12-week follow up (*p*<0.05), and proportion of ventilation volumes between 400-1000mls increased post intervention (*p*<0.001). A significant increase in the proportion of participants who “checked for responsiveness”, “opened the airway”, “performed a breathing check”, who used the “correct compression ratio”, and who used an “appropriate facemask technique” was also noted (*p*<0.001). A train-the-trainer model of resuscitation education was effective in improving resuscitation knowledge and skills in Sri Lankan rural peripheral hospital doctors. Improvement was sustained to 12 weeks for most components of resuscitation knowledge and skills. Further research is needed to identify which components of training are most effective in leading to sustained improvement in resuscitation.

## Introduction

Resuscitation education is an emerging field in Sri Lanka, run largely by consultant anaesthetists working at tertiary referral centers and the larger secondary hospitals. The heavy workload of these specialists limits the opportunity to provide training to rural peripheral hospital doctors. Previous research has identified that rural peripheral hospital doctors feel professional isolation due to a lack of training opportunities [1]. There is a need for good resuscitation knowledge and skills in these doctors as they are the first point of contact for patients who frequently require resuscitation such as those presenting with pesticide poisoning and snake bite injury [2–4]. A common emergency presentation and cause of death in rural Sri Lanka is organophosphorus pesticide (OP) self-poisoning, which is a condition that results in up to 200,000 deaths annually in Asia [5–9]. OP poisoning has a case fatality of 8-15% primarily due to respiratory arrest, which progresses to cardiorespiratory arrest [3,7]. Snakebite envenomations also require frequent resuscitation and high proportions (48%) of patients with neurotoxic symptoms who require mechanical ventilation have been reported [10,11]. 

At the time of this study advanced cardiac life support training (either according to the American Heart Association, UK or Australian resuscitation councils) was not nationally available for doctors. There are considerable logistic barriers in the delivery of training to doctors in these rural peripheral hospitals. Many peripheral hospitals are run by only one to two doctors, so they cannot be released from clinical duties to travel for 1-2 hours to the central hospitals where resuscitation education occurs.

The train-the-trainer (TTT) model of resuscitation education has been used successfully in higher resourced settings for various levels of resuscitation skills. For example, it has been successfully used to train university students to teach basic life support (BLS) but not advanced life support (ALS) [12,13]. The use of ‘non specialist’ trainers to teach resuscitation to doctors in teaching hospitals has also been previously described [14], however, the effectiveness of using peripheral hospital doctors as trainers in their own low resource rural hospital setting has not been examined. 

Those experienced in the global development of emergency medicine have advocated the TTT model of education to help leverage limited resources [15]. However, there have been no studies in the adult resuscitation education literature to date that have evaluated the effectiveness of this model by analysing validated knowledge and skills endpoints [16]. Two studies from Africa investigated the use of a TTT model but this was specifically for neonatal resuscitation [17,18], and another study commented on the success of the TTT approach in training nurses in cardio pulmonary resuscitation (CPR) in the Middle East, but did not objectively assess resuscitation skills in their course participants [19]. The only identifiable study that referred to a TTT model in either Sri Lanka or India was in the context of trauma care rather than cardiopulmonary resuscitation [20].

We sought a potentially sustainable solution to overcome the barriers to education and training for rural Sri Lankan peripheral hospitals by assessing the effectiveness of a TTT model that used non-specialist peripheral hospital doctors as trainers, who taught basic and advanced life support (ALS) training to their colleagues. Effectiveness was defined as a statistically significant improvement in components of resuscitation knowledge and skills towards an internationally recognised standard (based upon the International Liaisons Committee on Resuscitation - 2005 resuscitation guidelines).

## Materials and Methods

This study was approved by the Ethical Review Committee at the Faculty of Medicine, University of Peradeniya, Sri Lanka. As the study was carried out in conjunction with the regional government health training authority within the framework of usual practice, written consent was not required by the ethics committee. However as per protocol, trained research assistants described details of the study to participants and verbal consent was obtained prior to the commencement of the peripheral hospital workshop. Participant data was subsequently de-identified.

The study was conducted in the North Central Province of Sri Lanka between November 2008 and July 2009. The province has 45 peripheral hospitals with inpatient facilities supported by 2 central secondary referral hospitals. The TTT resuscitation training program was developed and conducted collaboratively with the North Central Provincial Department of Health. Our study population consisted of non-specialist doctors working at peripheral hospitals who were participants in the resuscitation training workshops and attended both the pre and post training assessments, including follow up at 6 and 12 weeks. 

### Training the trainers

The first phase of the study involved training of the trainers who consisted of 8 non-specialist doctors from five peripheral hospitals in the North Central Province of Sri Lanka. These 8 were selected from 20 candidates at an initial resuscitation workshop organized in conjunction with the provincial department of health, and were chosen because of their interest in becoming trainers and their observed competence. The prospective trainers each underwent two further sessions of residential training based at the tertiary teaching hospital for the province, which was centrally located (see Appendix S1 for an overview of the workshops). 

In the first one day session the candidates’ knowledge of the resuscitation syllabus was reinforced. In the second session, which was a two day ‘instructor workshop’, they were taught “how to teach” and how to run a peripheral hospital workshop (see Appendix S2 for schedule). The instructors who taught the prospective trainers were certified resuscitation instructors from schools of teaching affiliated with the International Liaisons Committee on Resuscitation (ILCOR). All instructors were experienced in teacher training according to either the ACLS (Advanced cardiac life support - American Heart Association standard) or ALS (Advanced life support - UK /European/Australasian resuscitation council standard) courses overseas. The manual for the course that the trainers were trained to teach was largely based upon the content of the UK resuscitation council’s “Intermediate ALS” course, which is also endorsed by the Australian and New Zealand resuscitation councils. 

The teacher training included adult education theory, how to run interactive lectures with digital video disc (DVD) support for content, and how to teach using a mannequin in skill stations and scenario stations. This session concluded with a practice workshop using volunteer participants who were junior doctors from the central hospital. The ‘trained trainers’ were directly observed and assessed on their ability to deliver lectures, run skills stations and conduct simulated scenarios according to a standardized checklist at this practice workshop (see Appendix S3 for checklists). 

A comprehensive instructor manual describing the workshop schedule and teaching goals was provided to the instructors and the trainers (see Appendix S3 for instructor manual). This also outlined the skills stations and scenarios being taught, and relevant adult education theory that would aid the delivery of course content. This manual also contained teacher assessment checklists that were used by the instructors to facilitate the feedback and assessment of the trainers, and allowed standardisation of the teacher training process. Our clearly defined teacher training methodology meant that outcomes measured later in the study could be linked to the effectiveness of teaching by the trainers, and thus the TTT model of resuscitation education being tested. The adult education theory and teacher training module in the manual was taken from the “Pocket guide to Teaching for medical instructors” by the BMJ group [21]. 

Prospective trainers from remote locations were accommodated in the same residential facility provided for by the course. This allowed time for orientation to the course goals, and for group reflection and consolidation of learning at evening meals, a situation that was likely to be different to their previous educational experience (see Appendix S2 includes schedule of workshop). This strategy was used in recognition of the time intensive nature of the instructor workshop, and in hope of supporting a team approach to learning that could increase esteem and self confidence during the process of ‘learning how’ to teaching. 

### Peripheral Hospital Resuscitation Workshops (Training Intervention)

The second phase involved sending the “trained trainers” to selected larger peripheral hospitals where they delivered eight resuscitation training workshops, teaching BLS and ALS, over a two month period (see Appendix S4 for course outline and contents). In this phase the resuscitation ‘Trainers’ worked in pairs to deliver education to 6-12 participants per session maintaining a minimum ratio of 1 instructor to 6 participants at all sessions. The participants were peripheral hospital doctors who were either from the same hospital where the trainers worked or who had travelled from a nearby smaller hospital. The aim of this workshop was to teach resuscitation to doctors who were assumed to have had no prior post-graduate resuscitation knowledge. The focus of the workshop was on the practical skills of BLS and ALS that would be required by these doctors in their daily practice, including an approach to the unresponsive patient, performance of chest compressions, and performance of ventilations using a bag valve mask apparatus, intubation and ventilation of an intubated patient. 

Participants of the peripheral hospital workshops were supplied with a resuscitation training manual, (which was a modification of the ALS manual for ‘intermediate life support’), and a wallet card with the cardiac arrest algorithm printed on it. Trainers carried out resuscitation education with the aid of training mannequins and other supporting equipment including a DVD containing video lectures, and wall charts of the resuscitation algorithm (see Appendix S5 for a list of supporting material for trainers and participants). Baseline data on the characteristics of the participants and their feedback on the training workshop were collected. Real resuscitation experiences that the participants encountered during the 12 week study period were also collected through a log sheet that was supplied to them at the beginning of the workshop. 

Each workshop was held from 0800hrs to 1645hrs, which included one and a half hours for multiple choice question (MCQ) testing and scenario testing, immediately before and after the resuscitation training intervention. The training intervention started with a series of lectures about BLS and airway management, which were pre-recorded and played from a DVD lasting approximately an hour. During this time the trainer would periodically pause the DVD and interact with participants about the content of the DVD lecture. A practical session teaching bag mask ventilation and intubation followed where each trainer worked with 6 participants in skills stations that were equipped with either a mannequin or an airway-training device. The practical skills were taught using Peyton’s 4-stage teaching approach as described in the European Resuscitation Council resuscitation course strategy [22]. 

The afternoon DVD lectures covered treatment algorithms for shockable and non-shockable cardiac arrest, and these skills were practiced in the same groups under the supervision of the trainer who made use of resuscitation mannequins to run two set scenarios. The trainers were required to give positive feedback and constructive criticism to participants during all the practical session including the scenarios. Posters of the cardiac arrest treatment algorithms were also supplied to each hospital that participants came from as part of the service component of the educational intervention.

The knowledge and skills of participants was assessed before and after they received resuscitation education from the trainers. This assessment was carried out by 3 research assistants, however, the workshop participants were taught exclusively by the “trainers”. The research assistants were all junior doctors and were trained and overseen by the principal investigator (PI). At 6 and 12 weeks participants were followed up with an identical assessment. Feedback on performance in the scenario was not given for the pre-training assessment, but provided for the post-training assessment, 6-week and 12-week follow ups. 

### Outcomes

The primary outcome was to assess the effectiveness of this model of teacher training in improving resuscitation knowledge and skill endpoints among the peripheral hospital doctors taught by the ‘trained trainers’. Knowledge and skills were judged by scores in an MCQ test and performance in a simulated cardiac arrest scenario with concurrent video analysis. Assessment was carried out immediately pre and post training, and again at 6 and 12 weeks, following the advice of international guidelines that recommended repeat assessments to ensure adequate retention of knowledge and skills following resuscitation training [23]. The secondary endpoint of this study was to investigate how long any improvements in knowledge and skills would be sustained.

### Knowledge assessment: MCQ test

The MCQ was a modified version of the American Heart Association (AHA) advanced cardiac life support (ACLS) course, which tested aspects of BLS and ALS including ECG arrhythmia recognition (see Appendix S6). One mark was awarded for every correctly answered question for each of the 30 questions, and no penalty was given for missed or incorrect answers. Five questions were modified to fit the Sri Lankan medical context of health care. For example, terms like “ER”, an acronym for ‘emergency room’ didn’t exist in the Sri Lankan health care terminology, so this was replaced with more appropriate terms such as “OPD” (outpatient department), or “ETU” (emergency treatment unit) for the written scenario statements that formed the basis of some questions. The same MCQ test was used at all assessments, but participants were not told their test scores nor were they given feedback on their answers to previous tests. 

### Skills assessment: performance in a cardiac arrest scenario

Participant’s knowledge and skills in the initial response to an unconscious patient, and performance of CPR was tested through a video recorded simulated scenario of cardiac arrest involving an instrumented mannequin [24]. 

A temporary ‘simulation suite’ was established in the room where the peripheral hospital training session was being conducted (see Appendix S7). The simulated patient was a male mannequin lying in a hospital bed wearing local attire, cordoned off from the rest of the room by hospital screens. The participant was read the scenario script from outside the simulation suite and was told that a 50 year old man had collapsed and the participant was asked to “do what you would do in real life” (see Appendix S7 for script). The participant then entered the suite and was expected to check the responsiveness of the patient, call for help, open the airway, perform a breathing check and after this assessment initiate single rescuer CPR. If CPR had not commenced by 60 seconds they were given the following prompt:-

“I want you to start cardiopulmonary resuscitation on this patient please”

The scenario was continued to capture 60 seconds of chest compressions once CPR had commenced and the participant was then told that help had arrived. 

### Assessment of compression and ventilations

The instrumented mannequin, Resusci Anne Skill Trainer™ (by Laerdal), was connected to a laptop, which recorded the following measurements of resuscitation skill;- ventilation count, adequate ventilation volume, average ventilation volume (milliliters), compression count, compression with no error, average depth (millimeters), adequate depth, compression rate, and adequate rate. We analysed the data by describing any changes in the raw scores of these variables in relation to the training intervention. We also described the changes in proportions of participants achieving clinically relevant benchmarks described in the literature reflecting the quantity and quality of compressions and ventilations. The benchmarks we reported were;- mean compression depth between 40-50mm, mean compression depth ≥ 40mm, mean compression rate 80-120/min, and mean ventilation volume 400-1000mls [25]. 

### Assessment of initial approach and steps of CPR

Video assessment of simulated scenarios is an established methodology in measuring effectiveness of resuscitation training [24–28]. A video recorded scenario was to measure objective endpoints on quality of compression and ventilations, and steps of cardiopulmonary resuscitation (CPR) employing validated metrics [24,25]. 

The video recorded scenarios were marked by 3 trained research assistants who coordinate the assessments at the peripheral hospital workshops. A marking schedule was adapted from a validated assessment tool for CPR performance called the ‘Cardiff Test version 3.1’ [24], and these variables were modified to be consistent with the 2005 ILCOR guidelines for CPR where necessary. The marking criteria assessed whether participants correctly carried out the following steps of initial approach and performance of CPR;- calls for help, checks for responsiveness (shouting), checks responsiveness (shaking victim firmly), opens airway (using either head tilt, chin lift or jaw thrust), checks for airway obstruction, performs a breathing check, uses correct compression ratio, and uses appropriate facemask technique (see Appendix S8 for marking checklist). The participants’ practice of checking for a pulse centrally, or utilising a precordial thump was also observed. 

The video assessors were trained on how to mark the video scenarios according to the marking schedule by the principal investigator (PI) in an initial 30 minute teaching session, Thereafter they independently marked 5 video recordings of scenario assessments, and a kappa score for inter-observer reliability of 0.69 was calculated for the variables that the study was reporting. This score was within the range of kappas reported by the original article for the Cardiff Test [24]. They subsequently received a second group training session where any discrepancies in marking were discussed and consensus was achieved. 

The remaining participant videos were then divided and allocated to each video assessor to mark independently. Participant’s sequential assessments were staggered between the three assessors, and they were blinded to their previous results when marking. The score from each assessment was recorded on a checklist, followed by subsequent data entry into an excel spreadsheet. The skills variables were analysed as binary outcomes where the task was either performed correctly or not. 

### Statistical analysis

A power calculation estimated a total of 50 participants were needed to provide more than 90% power to detect a statistically significant difference between the before and after composite scores using a two-sided paired t-test at significance level 0.05. This calculation was performed under the assumption that the performance scores would increase by an average of 15 points (from 50% to 65%) with a standard deviation of 30 points, based on observations from a previously conducted pilot study. The sample size calculation was performed using Stata v12. We aimed to recruit 75 doctors, to allow for attrition.

The mean MCQ scores were compared using a Wilcoxon signed rank test for paired non parametric data. Continuous variables were compared between assessment sessions using the repeated measures ANOVA test looking for a change in performance over time, and we have presented unadjusted *p*-values. Differences in proportions were tested using the McNemar exact test. Statistical tests were carried out using STATA v12, and graphs were created using GraphPad Prism (v6).

## Results

**Figure 1:**
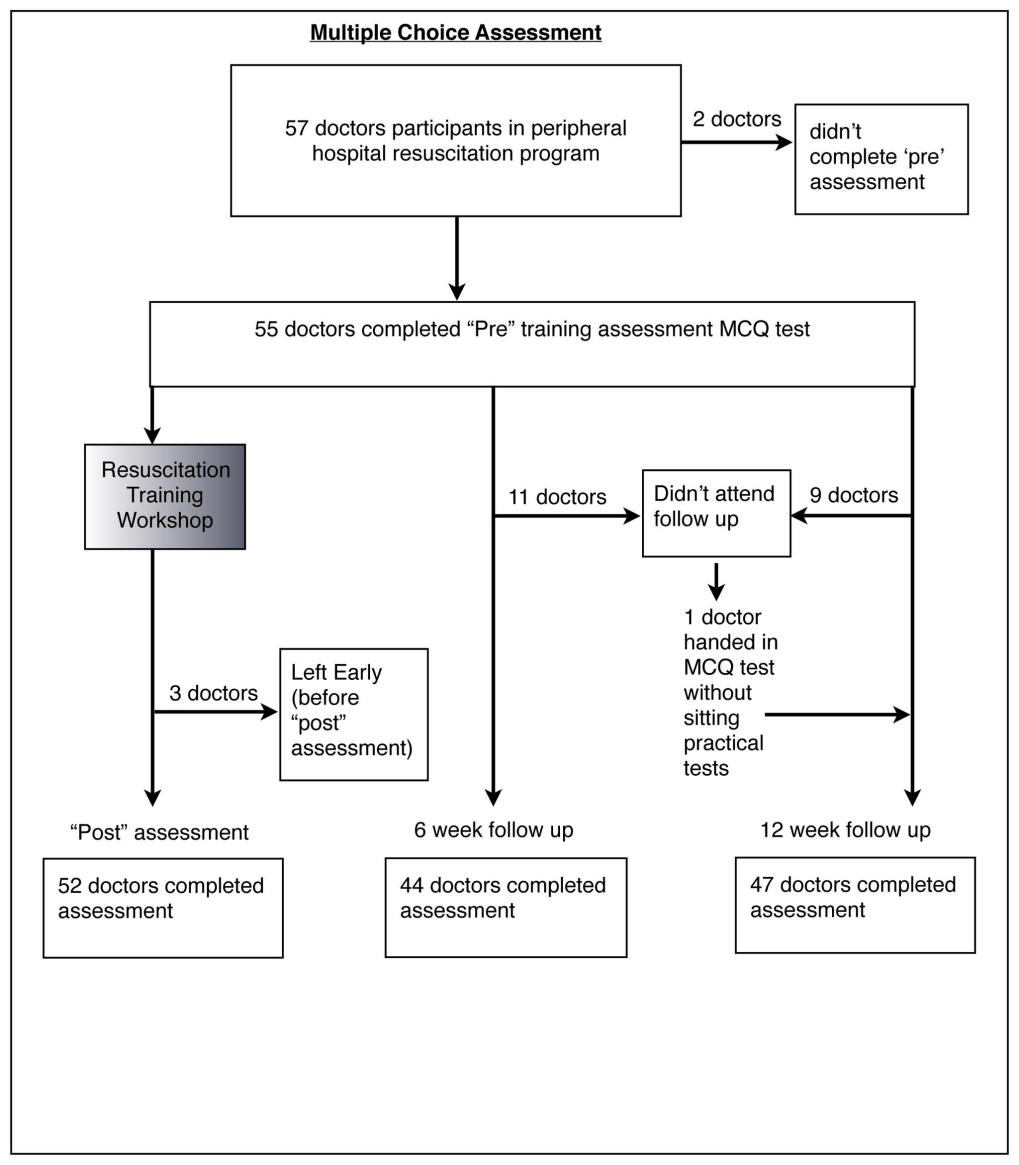
Flowchart showing the number of participants recruited to the study, receiving the training intervention and receiving follow up MCQ assessments.

**Figure 2:**
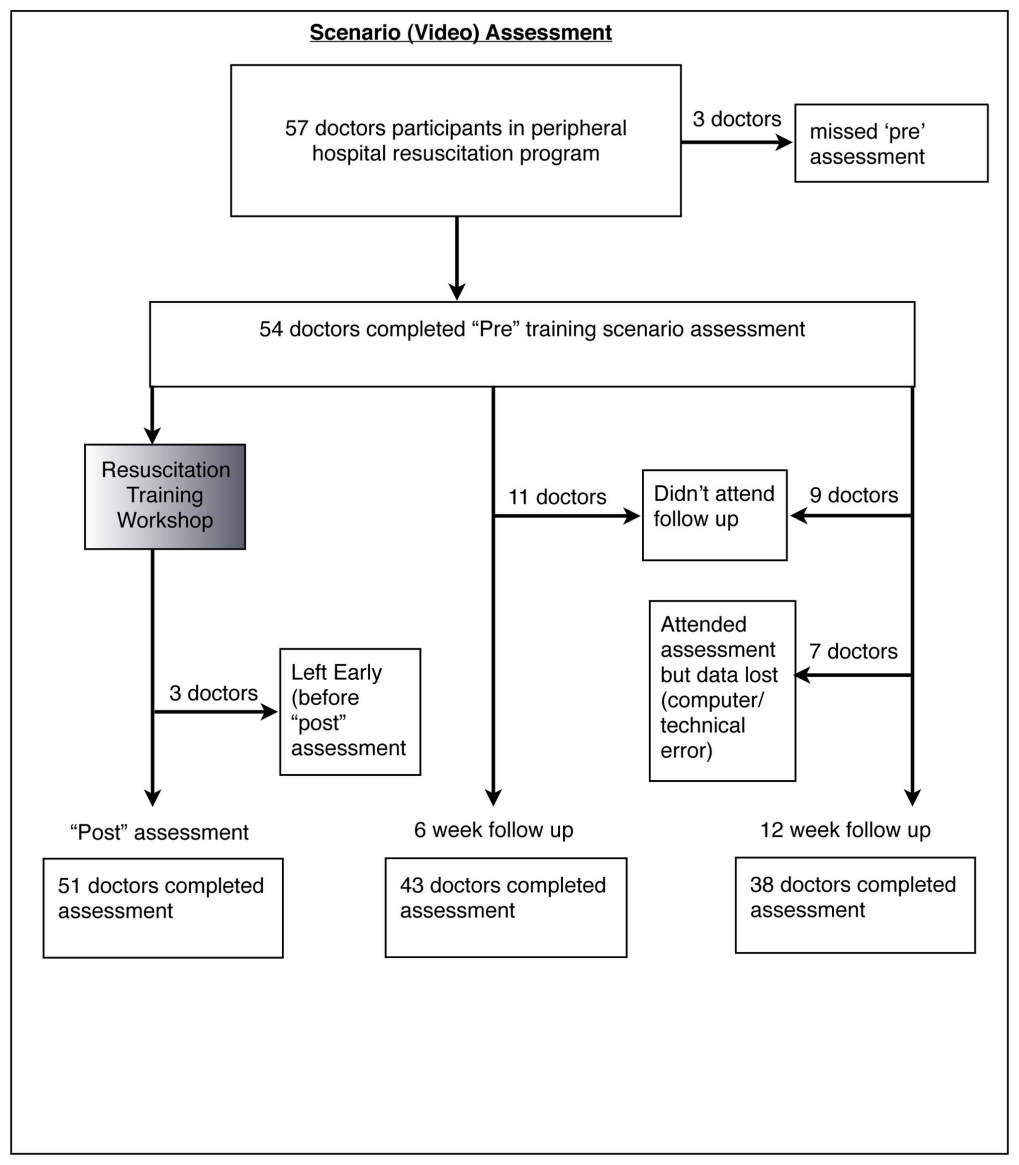
Flowchart showing the number of participants recruited to the study, receiving the training intervention and receiving follow up scenario assessments. The number of participants that did not attend follow up assessment and where there were technical errors leading to missing data is also shown.

57 participants attended the peripheral hospital workshops but two participants were excluded because they did not have a pre-intervention assessment leaving 55 participants for analysis. One participant did not complete the baseline data survey so demographic data was represented for only 54 participants (Table 1). Some participants were unable to sit the post-training and follow up assessments due to work commitments. 52, 44 and 47 participants received the post-training, 6 week and 12 week follow up MCQ assessments respectively (Figure 1). 51, 43 and 38 participants received the post training, 6 week and 12 week follow up scenario assessments respectively (Figure 2). In addition, data was lost for 7 participants in the 12 week follow up assessment due to a technical error involving the computer backup of data. A complete set of assessment data was available for 32 participants due to a combination of non-attendance for follow up assessments and missing data. 

**Table 1:** Demographics and baseline characteristics of the study population.

	***n=54 (%)***
***Age and Seniority***	
Median age (range)	34 (28-53)
Median years post graduation (range)	6 (2-28)
***Other characteristics***	
Male	39 (71%)
Any previous resuscitation education experience	14 (26%)
- Education included Scenarios	1 (2%)
- Education included Cardiac Rhythms	1 (2%)
Course attended within last 12 months	3 (6%)
Level of resuscitation training felt to be adequate	20 (37%)

The median age of participants was 34 years and there was a male predominance (39/55, 71%). The participants ranged from 2 to 28 years post graduation from their medical degree, and there was a median of 6 years postgraduate experience in the study group. 14/54 (26%) had experienced some form of previous resuscitation education, but only 3/54 (6%) experienced this education within the last year. Just one participant reported scenario training or interpretation of cardiac rhythms as a component of previous education. 3/14 (21%) from the subgroup reporting prior educational experience felt their resuscitation training was adequate, and overall 20/54 (37%) felt their resuscitation training experience was adequate prior to taking part in the study (Table 1). 

### MCQ Assessment

The mean MCQ score increased from 54.1% pre intervention to 66.6% post intervention, and 69.8% and 72.9 % at 6 week and 12 week follow ups, and this change over time was significant (p<0.001, Wilcoxon signed rank test) (Table 2). The repeated measures ANOVA also showed a difference in scores over time (p<0.001). 

**Table 2:** Mean scores and 95% confidence intervals are shown for MCQ assessment.

***MCQ Assessment***	**Pre-training, n=55**	**Post-training, n=52**	**6 weeks post, n=44**	**12 weeks post, n=47**	*p* value**
**Mean score**	54.1	66.6*	69.8*	72.9*	<0.001
**95% CI**	49.1-59.1	62.1 - 71.1	65.1-74.5	67.7-78.0	
**Median (p50)**	52	68	72	76	

Data was analysed with the non-parametric Wilcoxon signed rank test, and a significant difference between pre-training score and subsequent post-training assessments. The *p* value is also shown for the repeated measures ANOVA test which compared differences in mean scores over time.

^*^ *p*<0.001 Wilcoxon signed rank test

^**^Repeated measures ANOVA

### Scenario Assessment

The mannequin data showed a significant difference over time for 6 out of 9 variables related to performance of compressions and ventilations after analysis by the repeated measures ANOVA test. A significant change was noted for average ventilation volume, compression count, compression with no error, adequate compression depth, average compression depth, and compression rate (Table 3). The mean number of compressions almost doubled from 40 to 75.6, and compression with no error numbers improved from 2.5 to 19.2 when comparing pre with post (p<0.001), the latter variable reflecting the correct hand position, and depth considered together. 

**Table 3:** The effect of training on variables of resuscitation skills as recorded by the Laerdal mannequin.

***Category***	***Time Variable***	***Mean***	***p value***
**Ventilation count** (number of ventilations)	Pre-training	4.6	
	Post-training	5.2	
	6 weeks post	3.4	
	12 weeks post	3.3	0.213
**Adequate ventilation volume** (number of ventilations)	Pre-training	1	
	Post-training	2.1	
	6 weeks post	1.9	
	12 weeks post	1.7	0.134
**Average ventilation volume** (milliliters)	Pre-training	307.2	
	Post-training	458.8	
	6 weeks post	398.3	
	12 weeks post	342.5	0.004*
**Compression count** (number of compressions)	Pre-training	40.4	
	Post-training	75.6	
	6 weeks post	73.1	
	12 weeks post	70.4	0.001*
**Compressions with no error** (number of compressions)	Pre-training	2.5	
	Post-training	19.2	
	6 weeks post	14.3	
	12 weeks post	19.6	0.001*
**Adequate depth** (number of compressions)	Pre-training	4.9	
	Post-training	25.4	
	6 weeks post	21.9	
	12 weeks post	27.3	0.001*
**Average Depth** (millimetres)	Pre-training	28.8	
	Post-training	36.9	
	6 weeks post	37.1	
	12 weeks post	39.1	0.001*
**Compression rate** (compressions/minute)	Pre-training	106.1	
	Post-training	116.3	
	6 weeks post	107.1	
	12 weeks post	105.2	0.016*
**Adequate rate** (no of compressions)	Pre-training	46.9	
	Post-training	59.4	
	6 weeks post	75	
	12 weeks post	75	0.054

The *p* value was calculated using the repeated measures ANOVA test checking for a difference in mean performance over time.

**Figure 3:**
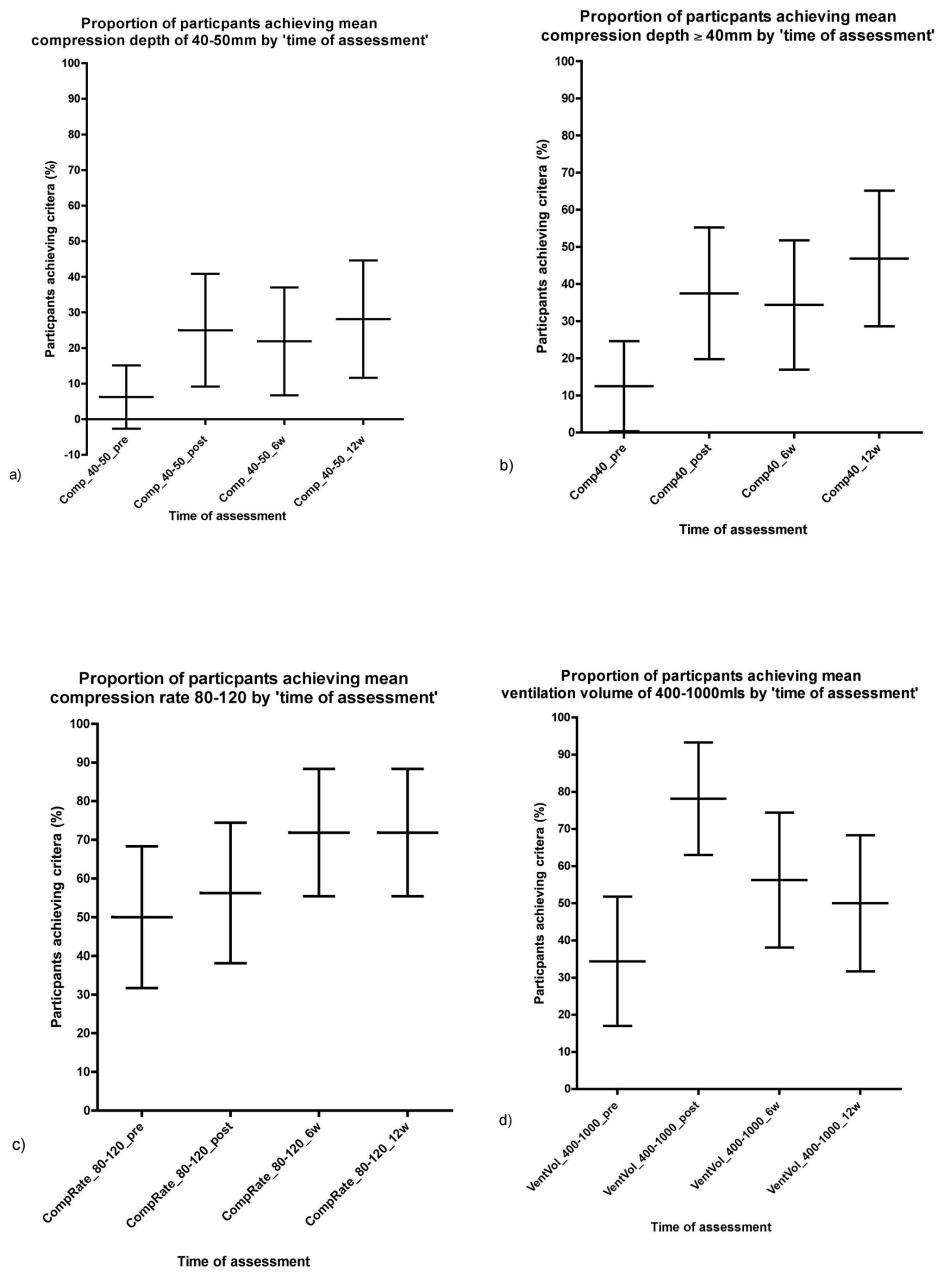
Graphs plotting percentage of participants achieving clinically relevant benchmarks (those achieving benchmark/total number in studied). a) mean compression depth of 40-50mm, b) mean compression depth ≥40mm, c) mean compression rate 80-120, and d) mean ventilation volume of between 400-1000mls. (The mean percentage has been plotted with error bar representing the 95% confidence of the mean) .

Clinically relevant benchmarks of compression and ventilation after the training intervention also improved. The proportion of participants with a mean compression depth ≥40mm (p<0.05) and proportion of participants with ventilation volumes between 400-1000mls (p<0.001) increased from the pre-training (baseline) to the post-training assessment (Figure 3, Table 4). There was a trend for sustained improvement at 6 and 12 weeks, for the “compression depth ≥40mm” variable, however, the improvement in proportions reached statistical significance only at the 12 week follow up. Conversely, the improvement in “ventilation volume between 400-1000mls” was not sustained beyond the post-training assessment. However, the proportion of participants achieving this benchmark at the 6 and 12 week follow up assessments was still greater than the baseline proportion (56% and 50%, versus 34%). Similarly a trend for increasing proportion for participants who achieved a “mean compression depth of 40-50mm”, and “compression rate of 80-120” was observed after the training intervention, but statistical significance was not achieved for these increases. 

**Table 4:** Percentage of participants appropriately performing aspects of chest compression and bag valve mask ventilation pre-training, immediately post-training and at 6 week and 12 week follow up.

	**Percentage performing (95% CI), n=32**			
	**Pre-training**	**Post-training**	**6 weeks post**	**12 weeks post**
Mean compression depth 40-50mm	6 (1-21)	25 (11-43)	22 (9-40)	28 (14-47)
Mean compression depth ≥40mm	13 (4-29)	38 (21-56)*	34 (19-53)	47(29-65)*
Compression rate 80-120 per minute	50 (32-68)	56 (38-74)	72 (53-86)	72 (53-86)
Ventilation volume 400-1000mls	34 (19-53)	78(60-91)**	56 (38-74)	50 (32-68)

^*^p<0.05

^**^p<0.001 p-values based upon a comparison of pre-training and post training intervention percentages using McNemar’s test (immediately post training, at 6 week follow up, and 12 week follow up)

95% exact CI were used

**Figure 4:**
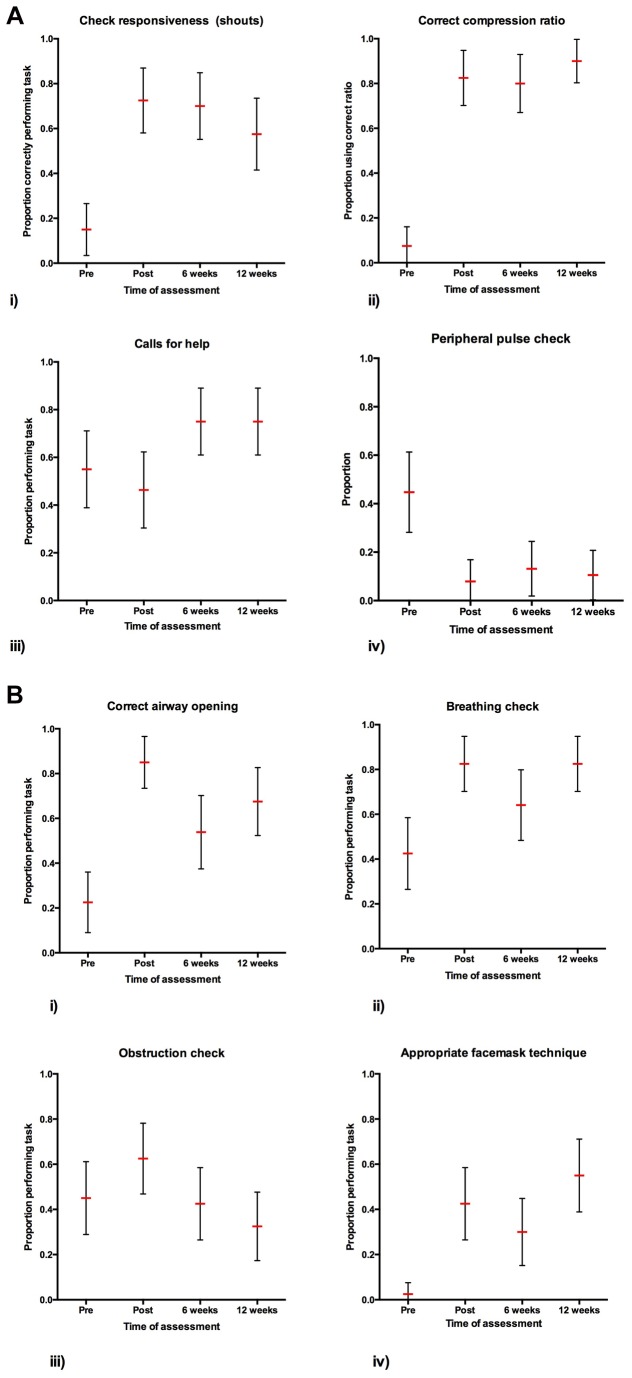
a) Shows proportion of participants carrying out the following responses from the video assessment component of the scenario (those performing task/total number studied); i) appropriately checking responsiveness by shouting, ii) using the correct compression ratio when performing cardiopulmonary resuscitation iii) appropriately calling for help, and iv) checking for the pulse only in a peripheral location (incorrect). b) Shows proportion of participants who correctly performed i) opening of the airway (either head tilt, chin lift or jaw thrust), ii) an obstruction check, iii) a breathing check, and iv) used an appropriate facemask technique (Nb - for Figures 4a and b the mean proportion has been plotted as a horizontal line with error bars showing the 95% confidence interval of the mean).

Results for the video assessment showed significant improvements in checking for responsiveness (shouts), airway opening, breathing check, performance of the correct compression ratio, use of an appropriate facemask technique, *p*<0.001 for all mentioned variables, (Figure 4a,b, Table 5). There was also a reductions proportions performing precordial thump (p<0.05), and checking the pulse at a peripheral location (p<0.001), which was not recommended. 

**Table 5:** Performance in video assessment variables before and after the training intervention.

**Video assessment variable**	**Mean percentage achieving variable in relation to training intervention, (*95% CI*)**			
	***Pre-training***	***Post-training***	***6weekspost***	***12weekspost***
Calls for help	55 (38, 71)	50 (28, 72)	73 (53, 94)	73 (49, 98)
Checks responsiveness - Shouts	16 (6, 31)	*77 (55, 98*)**	*69 (50, 87*)**	*58 (38, 78*)**
Checks responsiveness - Shakes	3 (0, 14)	5 (0, 13)	6 (0, 25)	14 (1, 26)
Pulse Check (all)	95 (82, 99)	79 (63, 96)	*66 (46, 86*)*	*66 (47, 85*)**
- Checks pulse (central location)	50 (33, 67)	71 (50, 92)	53 (30, 75)	55 (31, 80)
- Checks pulse (only peripherally)	45 (29, 62)	*8 (0, 28*)**	*13 (0, 31*)**	*11 (0, 30*)**
Airway Opening	18 (8, 34)	*84 (66, 100*)**	*50(29, 70*)**	*65 (42, 88*)**
- Head tilt	18 (8, 34)	*76 (58, 94*)**	36 (18, 55)	*63 (40, 86*)**
- Chin lift	8 (2, 21)	*66 (48,84*)**	*42 (24, 60*)**	*37 (17, 57*)*
- Jaw thrust	5 (1, 18)	*39 (18,60*)**	*26 (7, 45*)*	*26 (7, 45*)*
Airway obstruction check	47 (31, 64)	63 (38, 88)	44 (19, 69)	31 (5, 58)
Breathing check	45 (29, 62)	*82 (61, 100*)**	*61 (40, 82)*	*82 (61, 100*)**
Correct compression ratio	5 (1, 18)	*84 (66, 100*)**	*79 (62, 95*)**	*89 (75, 100*)**
Appropriate facemask technique	3 (0, 14)	*40 (22, 58*)**	*29 (13, 46*)**	*56 (36, 76*)**
Precordial thump	18 (8, 34)	*0 (0, 15*)*	*2 (0, 16*)*	*0 (0, 15*)*

^*^p<0.05

^**^p<0.001 p-values based upon a comparison of pre-training and post training intervention percentages using McNemar’s test (immediately post training, at 6 week follow up, and 12 week follow up).

The greatest proportional increases were observed for initial approach, where the proportion checking responsiveness by shouting “are you okay” increased from 16% pre intervention to 77% post intervention, and in CPR for “airway opening”, and “correct compression ratio”, increasing from 18% to 84%, and from 5% to 84% respectively. The improvement with the pre training scores remained significant at the 6 week and 12 week follow ups in the majority of variables as depicted in Table 5.

### Resuscitation Logbook

During the 12 week follow up period 31 participants (56%) reported between 1 to 4 real life resuscitation encounters in their hospital jobs (Table 6). They also reported that they found the training they received during the study intervention helpful in 91% of these encounters. 

**Table 6:** The number of real life resuscitation encounters reported in logbooks of study participants.

***Experienceofrealliferesuscitation***	**Number of Participants, n=55 (%)**
No reported resuscitation experience	24 (44)
Reported 1 or more resuscitation events	31 (56)
***Numberofresuscitationencounters***	
0	26 (47)
1	17 (31)
2	5 (9)
3	7 (13)
4	2 (4)
***Eventswheretrainingwashelpful***	**Number of events, n=57 (%)**
Yes	52 (91)
No	5 (9)

## Discussion

The current study tested a novel approach to resuscitation education that has not been previously reported in the literature. Whilst there are other studies that measure the benefit of different educational interventions in improving ALS and BLS outcomes in highly resourced urban settings, the key difference with our study was its focus on the assessment of a train-the-trainer (TTT) model of education using non-specialist doctors in a resource limited peripheral hospital setting. We also reported objective and validated endpoints in resuscitation knowledge and skills, directly after and up to 12 weeks following the training intervention. Our study has direct relevance to rural Asia where there is a high incidence of respiratory failure from pesticide poisoning and snake bite envenomation [4,5,10,11], in addition to the standard range of primary cardiorespiratory pathology seen throughout the world.

The main objective of our training intervention and evaluation was to improve outcomes in accordance with the recommendations set out by ILCOR. These guidelines emphasised high quality chest compressions with minimal interruptions, and recommended a cardiac compression to ventilation ratio of 30:2 and a target chest compression rate of 100 per minute [29]. 

We observed improvement in all the domains of data we collected including knowledge from the MCQ scores, quality of compressions and ventilations from the mannequin data, and knowledge and skills in carrying out the “steps of CPR” through the video assessment. The improvement seen in these metrics was similar to reports of comparable endpoints from training interventions delivered in high resourced clinical environments [25,30,31]. 

### Improvement in Compressions and Ventilations

The improvements we noted in compression and ventilation skills, as recorded by the instrumented mannequin, were significant, but in general not as large as that observed for the “steps of CPR” as measured by the video analysis. The most prominent improvements were compression counts (number of compressions), and compression depth (including adequate depth), which both significantly improved after the training intervention. The increases in endpoints from the instrumented mannequin data also translated into significant improvement in clinically relevant benchmarks, specifically in the proportion of participants achieving compression depths of ≥40mm, and ventilation volumes of 400-1000mls (Table 4). However, the absolute proportions of post intervention compressions depth was suboptimal, reflected by low absolute proportions (38 - 47%) of participants achieving ≥40mm depth, despite improving significantly from the baseline proportion (13%). 

Suboptimal chest compression depth is a recognised challenge in ALS training and other studies have reported similar, or lower, chest compression depths than reported in the current study despite being conducted in high resourced settings [30–32]. Perkins et al. studied the quality of CPR achieved by health care professions attending ALS training courses in the UK, and reported mean chest compression depths of between 24.1 - 28.2mm [30], whereas the mean compression depth in our study increased from a baseline 28.8mm to between 36.9 - 39.1mm post training. Two studies identified that poor chest compressions depths in mannequins placed on hospital beds may be due to pressure dissipation through the mattress rather than due to lack of chest compression skill [31,32]. Another study reported higher proportions (70-79%) of participants achieving an adequate compression depth (≥40mm) [25], however, it is likely that the mannequin they used was placed on the floor which may have aided their compression depth recordings [32]. 

While there was no increase in the “ventilation counts” there was a significant increase in the “volume of ventilation”, and the proportion of participants who delivered a clinically acceptable ventilation volume, i.e. 400-1000mls when comparing baseline with post intervention assessments (Tables 3 and 4). The proportion of participants who delivered an acceptable ventilation volume following training increased from 34% to 78%, this was higher than that reported by Mpotos et al. who reported between 52-59% of participants achieving the same following training intervention [25]. 

The skills that improved the most were use of the correct compression: ventilation ratio, correct airway opening, and delivery of adequate ventilation volume. The improvement in correct compression ratio is notable because it was in alignment with the CPR guidelines taught at the peripheral hospital workshops. The observed improvements in ventilation and airway opening skills were of particular importance in the study setting of rural Sri Lanka because airway compromise is commonly encountered due to the high incidence of organophosphorus poisoning and snakebite injury in the region [3,4,7,8,11,33].

The skills that improved the least were calling for help, checking responsiveness through shaking the victim, and checking for an airway obstruction. The latter two variables were difficult to measure through video analysis and are known to have a lower inter-observer reliability than for other measured variables measured [24]. 

### Retention of knowledge and skills over time

The retention of knowledge and skills at 6 weeks and 12 weeks, was a secondary endpoint in our study, and we noted that the mean post-training MCQ scores stayed elevated compared to the baseline assessment scores, and the improvements in most categories of the video assessment evaluating correct steps of CPR were also sustained until 12 weeks. However, when looking at the skills of chest compression and delivery of ventilations, only improvement in chest compressions (mean compression depth ≥40mm) was sustained until 12 weeks. The percentage achieving ventilation volumes of 400-1000mls dropped down from 78% at the post intervention assessment to 56% and 50% at the 6 and 12 week follow up assessments respectively. This suggests that the improvement in ventilation skill was not significantly sustained beyond the post intervention assessment. 

We also observed non statistically significant trends for increasing outcomes at the 6 week and 12 week follow up assessments for MCQ score, at the 12 week follow up for “initial airway opening” and “initial breathing check”, and at the 6 week follow up for “mean compression rate of 80-120” (Figure 3, and Figure 4b). One would expect a decrease in skill outcomes the greater the time from training intervention, so these trends were unexpected. In search of a plausible explanation we considered the possibility of continued learning following the initial training intervention, and the possibility of the follow up assessments having an educational benefit. Kromann et al. showed testing as a final activity in a resuscitation skills course increases learning outcomes (a so called “testing effect”), however, the effect of multiple testing without preceding formal training was not studied [34,35]. Our study was not designed to evaluate the role of repeat testing, but it would be worthwhile asking this question in future research investigating what factors lead to a sustained improvement of knowledge and skills in the peripheral hospital setting. 

This study confirms previous reports of a deficit in resuscitation training in the rural Sri Lankan setting [1]. Whilst 14 participants (26% of study group) had received previous resuscitation education (and only 6% had attended one within the previous year), none had received education equivalent to the standard of an ALS course. We know this because ALS training was not available for rural hospital doctors at the time of the study intervention. However, it was also confirmed through the participant data where only one participant reported prior exposure to scenario based education and education about cardiac rhythms as part of their previous resuscitation training experience (Table 1), which is an essential characteristic of ALS training. The lack of equivalency between the previous resuscitation education that was reported by participants and the education provided in the current study meant that prior training was unlikely to be a confounding variable in our analysis. In addition, the fact that 57% of the entire group, and 79% of the 14 participants had received previous resuscitation education, felt their level of resuscitation training was inadequate prior to the study intervention, perhaps provides further evidence of the magnitude of the resuscitation training deficit that exists in the peripheral hospital setting. 

In contrast to other studies reporting resuscitation training interventions, which are often set in urban teaching hospitals, our study delivered training to quite a diverse range of doctors who had between 2 and 28 years of post graduate experience. The heterogeneity of clinical experience within the study group, suggests a range of different learning aptitudes, and it is possible that this diversity could have been a challenge for the training model, and may have impaired the effect of the training intervention. It would be useful for further research to investigate the impact diversity of age and clinical experience on learning and teaching future studies in similar settings, as this appears to be a feature of rural doctor populations. 

The logbook data showed that 54% of participants were involved in real resuscitations over the 12 week follow up period and that they considered the training intervention helpful in a large majority (91%) of these events (Table 6). A subgroup analysis of those participants who reported real life resuscitation exposure is limited by the low numbers of resuscitation events per participant, even though a relatively high proportion of the group experienced real-life resuscitation. Furthermore, a meaningful correlation of real life exposure with the study endpoints would be flawed given that many of the resuscitation events occurred at different times in relation to the follow up assessments. In addition there was no unified experience with exposure as participants reported a number of different cardiac arrest scenarios ranging from myocardial infarction, snakebite, electrocution, trauma to intentional overdose (data not presented in results). Whilst a detailed analysis of the logbook data was beyond the scope of the current study, further research investigating the impact of real life resuscitation exposure on the learning and retention of resuscitation skills would be valuable in further developing course content. This is of particular relevance to the rural developing world setting, where the most common causes of cardiac arrest are likely to differ from the countries from which most of the clinical evidence behind the ALS courses originates. 

### Study limitations

As our study did not have a control arm there was the potential for participants to self learn between the intervention and assessment. However, improvement in the assessment immediately post intervention, when compared with the baseline assessment, strongly supports the finding that the study intervention was effective in this setting. It is conceivable that learning independent to the training intervention, could have affected the 6 and 12 week follow up assessments, but this was unlikely given the lack of institutional resuscitation training routinely available to participants in the study setting. Another study that did not have a control arm, reported a study design that involved testing immediately after the training intervention similar to that of our study [36]. By contrast, other studies where there was either no baseline [25,37,38] or a long interval between the baseline and post training assessment [34,35,39], a control arm was necessary because candidates could conceivably practice or learn independent to the training intervention. Nevertheless, we would recommend a randomized controlled trial in particular for research whose primary focus is on retention of resuscitation skills. 

The use of the same MCQ test in each assessment was a potential limitation as it is possible that repeated testing may have contributed in part to the increased scores that we attributed to increased theoretical knowledge. However, we attempted to minimise the learning of specific answers by not providing feedback to participants after they sat their MCQ tests. The alternative of using different MCQ tests for repeated assessment has reported limitations related to a lack of equivalency between tests [40]. A study by Ringstead et al. attempted to validate the reliability of different tests, and found a small, but significant, difference (4%) between MCQ tests scores that were designed by an expert panel from The European Resuscitation Council, and thus suggested caution should be used in interpretation of learning outcomes from different tests [41].

Missing data resulting from non-attendance by some participants for the follow-up assessments also limited our study. The most common reason for non-attendance was participants working as peripheral hospital doctors in situations of relative professional isolation with limited back up for clinical duties, precluding them from attending training sessions, a problem that has been identified in previous research [1]. Three participants were lost to follow up due to personal health factors; two participants became pregnant, and one participant developed chronic illness. Financial pressures were also a factor as many rural hospital doctors would often work a second clinical job that started in the late afternoon and evening, sometimes close to the time when follow up assessments were being conducted. This same issue affected the trainers themselves who devoted the most personal time toward this training intervention out of all the peripheral hospital doctors involved in the study. It is also possible that follow up assessments themselves were initially viewed as less educationally valuable than the resuscitation training workshop, which could have accounted for the initial drop in numbers between the post training assessment and the 6 week follow up assessment. However, the positive feedback that participants provided and the lack of increasing numbers of non-attendees in the 12 week follow up (Figure 2) suggests that participants may have seen benefit in the follow up assessment despite their work challenges, after attending one and understanding what it entailed. 

Data from the instrumented mannequin was missing in 8 participants and this also contributed to the study limitations. Stored data was lost from the computer in 7 participants due to a problem with backing up of laptop data, and data was lost from the mannequin itself in 1 participant due to a power failure at the peripheral hospital where testing was being conducted (lasting 30 mins). These technical errors were not easily avoidable given the context of “remote testing” in a rural part of a developing world country, and given our experience we recommend that any research using mobile simulation devices in a similar setting should employ a robust strategy for maintaining mannequin power supply and ensuring adequate computer storage of mannequin data. 

Despite these challenges the missing follow up data did not affect the comparison between the pre and post intervention which lead to the main conclusions of the study, and it also did not preclude us from making statistically significant findings regarding the sustained improvement of resuscitation knowledge, and some aspects of resuscitation skills, up to 12 weeks following the training intervention. Nevertheless, the problems of non-attendance and technical difficulties illustrate the challenges of conducting a study in a resource limited rural setting. In the same vein, other studies looking at resuscitation education in similar contexts have argued the value of research being based in a “typical” setting despite the challenges associated with it [42]. Other researchers reporting methodology guidelines for studies introducing complex interventions have also suggested that researchers need to carefully consider the trade off between the “importance of the intervention” and the value of the evidence that can be gathered given the constraints [43]. In accordance with this message we considered that the limitations of conducting research in our study setting (such as needing to use a mobile simulation suite rather than testing participants in a simulation centre that was absent in the region) may have been outweighed by the gaining a real life practical analysis of the intervention in question. 

The scenario we used was validated for initial approach and cardiopulmonary resuscitation [24,25]. However, it was not sensitive enough to capture the assessment of all the components of ALS such a specialised resuscitation treatment algorithms (such as ventricular fibrillation), some other areas of decision making, and team leadership. Some other studies coming from teaching hospital settings have used more complex metrics, involving more then one scenario where participants also worked in teams, but these metrics relied upon experienced ALS instructors to be assessors [36,39,41,44]. Such an approach would not have been possible in the rural setting that our study was conducted, as access to experienced ALS instructors was not possible for the duration of the study follow up. We chose to use a single video recorded scenario on an instrumented mannequin rather than multiple scenarios that were assessed real time because this approach had been validated [24], and was more practically suited to our study setting. 

### Future research directions

There have been no studies that compare and contrast the delivery of a standard ALS course though the TTT model of training, with other methods of resuscitation training. Self-learning using multimedia and other educational technology are becoming increasingly employed as strategies in resuscitation training, with the advantage of learner flexibility, and the (unproven) suggestion of cost effectiveness [25,36,38,45]. However, there have been mixed results from this approach in the context of ALS training and it has been suggested that face to face learning is unlikely to be replaced by education technology [38,45]. One study of ALS training yielded positive resuscitation outcomes from a multimedia learning strategy compared with reading alone [36], and another RCT evaluating e-learning failed to show improvement in objective outcomes despite positive evaluations by students of the course. These studies were all conducted in urban teaching hospital settings, in contrast to our resource limited study setting, where training hardware (including computers and resuscitation mannequins) were lacking. The effectiveness of these newer educational interventions needs to be assessed against current benchmarks for training in various clinical and resourced settings. 

### Train the trainer resuscitation education: complex intervention

The implementation of a train the trainer system of education in a rural resource limited setting represents a complex intervention because the infrastructure necessary to conduct a standard resuscitation education course does not exist in the same way that is present in an urban teaching hospital environment. We recommend that our results should also be interpreted in the context of the complex intervention that occurred which were probably essential for the results we observed. The in-depth teacher training component described in the methods and appendices, and collaboration with a local training authority (the Provincial Department of Health), which allowed the appropriate leave and provision of residential training for the trainers, as well as provision of a course manual, and wallet card and poster visual aids were either directly or indirectly part of our study intervention and therefore likely to be linked to the results we observed. However, the relative importance of these non-core training activities in achieving these results remains a topic for further process-based research to differentiate. For instance, the same course taught without the same level of emphasis on teacher training, or same support and collaboration offered by the local training authority may not have achieved the same results. Thus whilst we can be confident of our findings in the setting in which they were studied, we suggest that policy makers and researchers pay attention to the detail contained within the methodology of the “teacher training” and “assessment” components. We also suggest that the systems and processes employed in the use of this model of train-the-trainer resuscitation education be taken into account when planning and developing future related research and educational policy. 

## Conclusions

We found that the train-the-trainer model of resuscitation education reported in this study, which used non-specialist trainers, was effective in improving resuscitation knowledge and skills amongst peripheral hospital doctors in Sri Lanka. Furthermore many variables of assessment showed improvement that was sustained for up to 12 weeks post training intervention. Further research investigating the components of a training course that lead to improved knowledge and skills retention would be of benefit in developing effective resuscitation education programs for rural developing world emergency care systems. 

## Supporting Information

Appendix S1 **Resuscitation training intervention – overview of workshops.**
(DOC)

Appendix S2 **Instructor workshop (‘training the trainers’) – course outline.**
(DOCX)

Appendix S3 **Resuscitation Training “Instructor Manual”.**
(DOC)

Appendix S4 **Peripheral hospital resuscitation workshop – course content and outline.**
(DOC)

Appendix S5 **Supporting material for trainers & checklists of performance.**
(DOC)

Appendix S6 **MCQ test used at assessments.**
(DOC)

Appendix S7 **Script for resuscitation scenario & picture of assessment room.**
(DOC)

Appendix S8 **Marking schedule for video assessment.**
(DOCX)
