# Supplementary material for: The Effectiveness of a ‘Train the Trainer’ Model of Resuscitation Education for Rural Peripheral Hospital Doctors in Sri Lanka
Source: PLoS One. 2013 Nov 8;8(11):e79491. doi: 10.1371/journal.pone.0079491 (PMC3821851; doi:10.1371/journal.pone.0079491)
Supplement: Appendix S1 — Resuscitation training intervention – overview of workshops. (DOC) [file pone.0079491.s001.doc]

# Resuscitation Training Workshops;

### Overview of Workshops

| 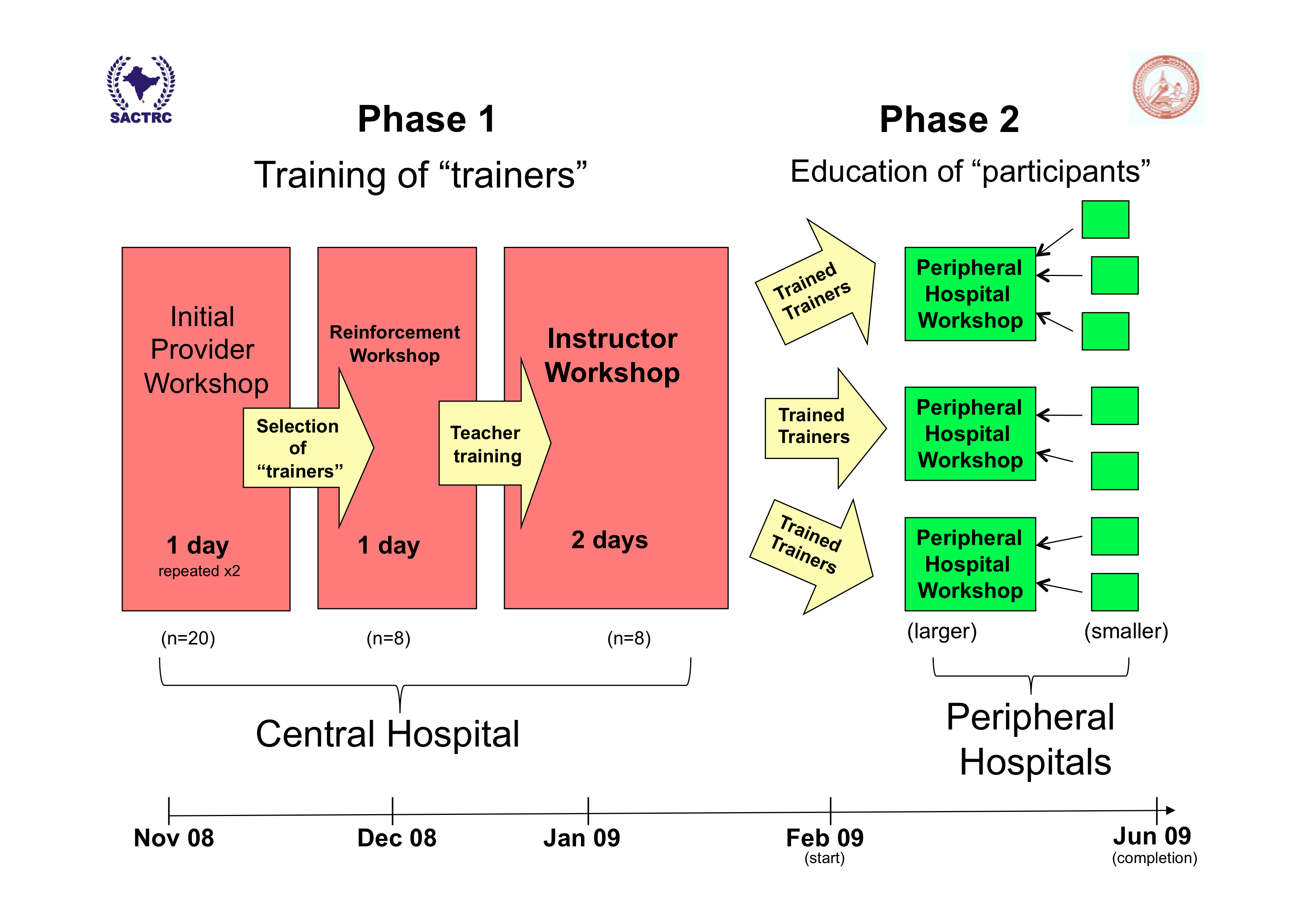 |
| --- |

### Overview of assessments

| 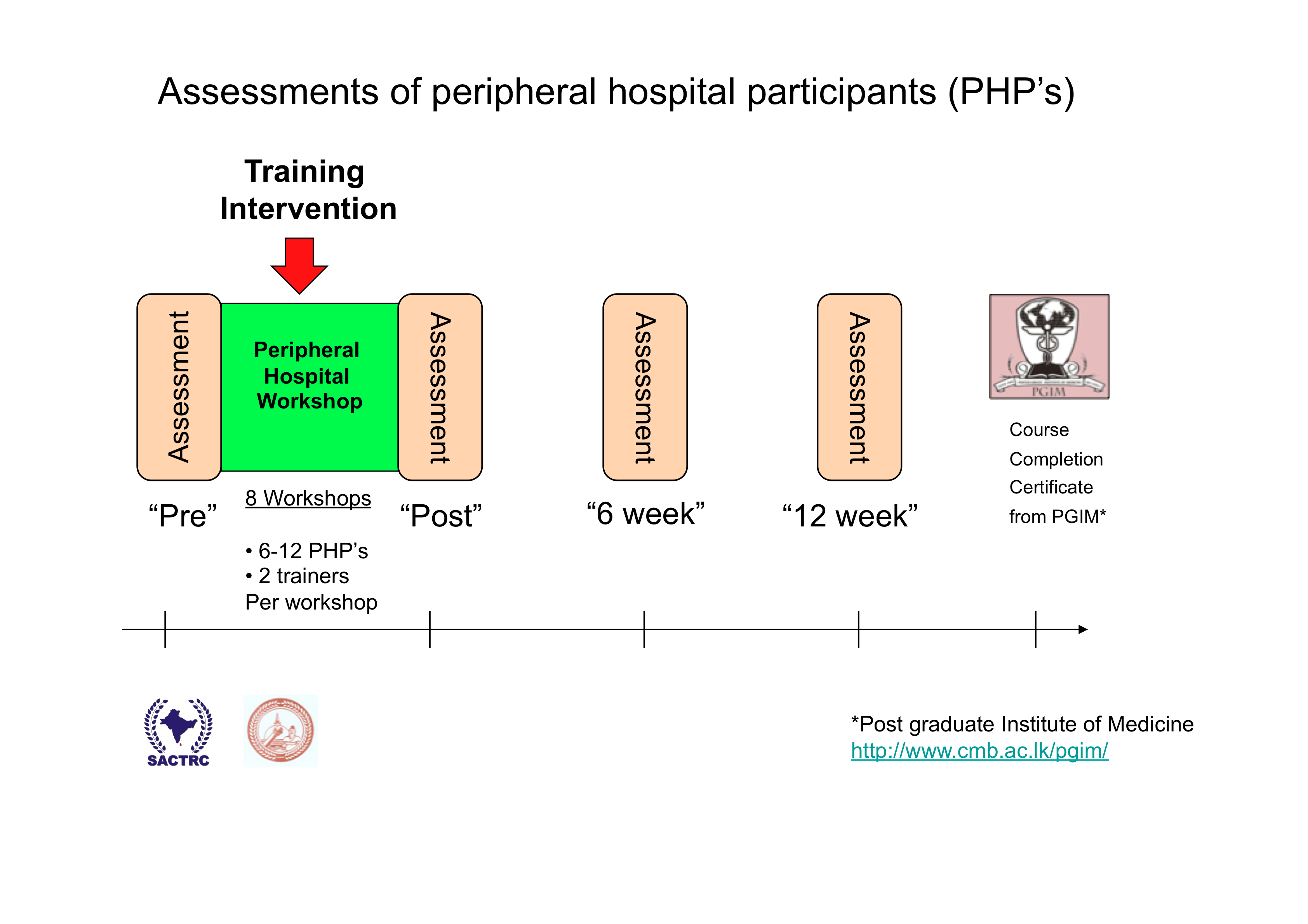 |
| --- |

# Phase Ia - Initial Resuscitation Workshop – Provider course & selection of trainers, Nov 28^th^ – 29^th^ 2008

Pre course Inauguration for Consultants and Day 1 participants

|  |  |  |  |
| --- | --- | --- | --- |
| ***Friday 28th November - Innauguration and Dinner (Nuwaraweva Resthouse)*** | | | |
|  |  |  |  |
| 19:00 |  | Registration |  |
| 19:30 | 10 mins | Welcome of distinguished guests and participants | Dr Athupattu, Provincial Director of Health Services – North Central Province (NCP) |
| 19:40 | 20 mins | Partnerships with provincial authorities to improve health care service delivery in the peripheries of NCP | Professor Andrew Dawson – South Asian Clinical Toxicology Research Collaboration (SACTRC) Program Director |
| 20:00 | 20 mins | Introduction and objectives of the resuscitation training program | Dr Bishan Rajapakse “Resuscitation Training” project coordinator |
| 20:20 | 15 mins | Keynote delivery on the "Importance of the development of the health system in the peripheries" | Hon Peshara Jayarathne - Provincial Minister of Health - NCP |
| 20:35 | 10 mins | Academic Agenda for the resuscitation workshop | Dr Bishan Rajapakse “Resuscitation Training” project coordinator |
| 20:45 | 5 mins | Words from the Peripheral hospital doctors | Nominated Peripheral hospital doctor |
| 20:50 | 10 mins | Vote of Thanks | Dr Dhammika De Silva, Medical Officer Planning, Office of the Provincial Director of Health Services (PDHS) |
| 20:40 |  | **Dinner** | |

Day 1 – resuscitation program (first 10 participants)

| ***Saturday 29th November - Workshop session 1 (Auditorim RDHS)*** | | | |
| --- | --- | --- | --- |
|  |  |  |  |
| 7:30 | 20mins | Introduction - Overview and prevention of cardiac arrest | **Dr Prasanga Palihawadana** . (Consultant Anesthetist, CA) |
| 7:50 | 40mins | Lecture 1 - Airway management, ventilation and Intubation | **Dr Prasanga Palihawadana** (CA) |
| 8:20 |  | ***Morning Practical Session 1 (rotate between stations)*** | |
| Simultaneously run (25 mins each station - 5 mins change time) | | Station A1 & A2 | Basic Airway (airway opening techniques, adjuncts, BVM ventilation) |
|  |  | Station B1 & B2 | Advanced Airway (intubation) |
| 9:15 | 45mins | Lecture 2 - "Advance Life Support Algorithm" | **Dr Asoka Gunaratne** (CA) |
|  | 30mins | Lecture 3 - "Tachycardia, Bradycardia algorithm" | **Dr Colin Page** (Emergency Physician, EP) |
|  | 20mins | ***Demonstration Scenario*** | **Consultant trainers and some participants** |
| 10:50 | 15mins | ***Morning Tea Break*** | |
| 11:05 | 20mins | Lecture 4 – “Post Resuscitation Care” | **Dr Lushantha Padmasiri** (CA) |
| 11:25 | 2 hours | ***Practical Session 2 - rotate through 4 skills stations*** | |
| Simultaneously run (25 mins each station - 5 mins change time) | | 1- Asystole/PEA scenario | **Dr Prasanga Palihawadana** (CA) |
|  |  | 2- VT/ VF scenario | **Dr Asoka Gunaratne** (CA) |
|  |  | 3- Tachycardia/ Bradycardia Scenario | **Dr Colin Page** (EP) |
|  |  | 4 - Post resuscitation care scenario | **Dr Lushantha Padmasiri** (EP) |
| 13:30 | 1 hour | **Lunch Break** | |
| 14:30 | 45mins | Post test - MCQ | Classroom |
| 15:15 | 1 hour | ***Post test practical Scenarios*** | (Conducted in Two places simultaneously) |
|  |  | ***Afternoon Tea*** *(refreshments served in between assessments)* | |
| 16:30 | 10mins | Closing comments | Entire Group |
| 16:40 | 30mins | Instructor de-briefing session | Nuwaraweva Resthouse |
| 17:00 |  | Instructors - Free time |  |

Pre-course introduction for Day 2 participants

| ***Sat 29th November - Welcome Dinner: Sunday participants (Nuwaraweva Resthouse)*** | | | |
| --- | --- | --- | --- |
|  |  |  |  |
| 20:00 | 5 mins | Introduction and aims of “Train the Trainer” project | Dr Bishan Rajapakse - project co-ordinator |
| 20:05 | 5 mins | Official Welcome to peripheral hospitals doctors | Dr Dhammika De Silva, MO Planning office of the PDHS, NCP |
| 20:10 | 15 mins | Guest Lecture - "The importance of resuscitation in the peripheral hospital setting" | Dr Herath (Anuradhapura – CA) ***or*** other Consultant Trainer |
| 20:35 | 5 mins | Words from the Peripheral hospital doctors | Nominated Peripheral hospital doctor |
| 20:40 |  | **Dinner** | |

Day 2 – (repeat course for day 2 participants, another 10, total of 20 receiving course over 2 days)

| ***Sunday 30th November - Workshop session 2 (Auditorim RDHS)*** | | | |
| --- | --- | --- | --- |
|  |  |  |  |
| 7:30 | 20mins | Introduction - Overview and prevention of cardiac arrest | **Dr Prasanga Palihawadana** |
| 7:50 | 40mins | Lecture 1 - Airway management, ventilation and Intubation | **Dr Prasanga Palihawadana** |
| 8:20 |  | ***Morning Practical Session 1 (rotate between stations)*** | |
| Simultaneously run (25 mins each station - 5 mins change time) | | Station A1 & A2 | Basic Airway (airway opening techniques, adjuncts, BVM ventilation) |
|  |  | Station B1 & B2 | Advanced Airway (intubation) |
| 9:15 | 45mins | Lecture 2 - "Advance Life Support Algorithm" | **Dr Asoka Gunaratne** |
|  | 30mins | Lecture 3 - "Tachycardia, Bradycardia algorithm" | **Dr Colin Page** |
|  | 20mins | *Demonstration Scenario* | **Consultant trainers and some participants** |
| 10:50 | 15mins | ***Morning Tea Break*** | |
| 11:05 | 20mins | Lecture 4 – “Post Resuscitation Care” | **Dr Lushantha Padmasiri** |
| 11:25 | 2 hours | ***Practical Session 2 - rotate through 4 skills stations*** | |
| Simultaneously run (25 mins each station - 5 mins change time) | | 1- Asystole/PEA scenario | **Dr Prasanga Palihawadana** |
|  |  | 2- VT/ VF scenario | **Dr Asoka Gunaratne** |
|  |  | 3- Tachycardia/ Bradycardia Scenario | **Dr Colin Page** |
|  |  | 4 - Post resuscitation care scenario | **Dr Lushantha Padmasiri** |
| 13:30 | 1 hour | **Lunch Break** | |
| 14:30 | 45mins | Post test - MCQ | Classroom |
| 15:15 | 1 hour | ***Post test practical Scenarios*** | (Conducted in Two places simultaneously) |
|  |  | ***Afternoon Tea*** *(refreshments served in between assessments)* | |
| 16:30 | 10mins | Closing comments | Entire Group |
| 16:40 | 30mins | Instructor de-briefing session | Nuwaraweva Resthouse |
| 17:00 |  | Return to Colombo |  |

# Phase 1b - Reinforcement workshop for “trainers” (n=8), 21^st^ December 2008

Overview of Training for subsequent workshops:

Nov 28-30 Phase Ia Initial Resuscitation workshop

Dec 21 Phase Ib (reinforcement workshop) – current

Jan 16-18 – Phase Ic workshop (How to be a trainer)

February Phase II resuscitation training at peripheral (‘hub’) hospitals

### Agenda for Workshop

7:45 – 8:15 Pre-Test

8:15 – 9:45 Review of resuscitation material & pre-test

9:30 – 10:00 Morning Tea

10-13:00 Skills stations (x3)

- Airway management (approach to an unresponsive patient & Intubation)

- Megacode station 1

- Megacode station 2

13:00- 14:00 Lunch

1:45 – 15:00 Further scenarios based on Lunchtime feedback

# Phase Ic – “Instructor workshop”, n=8

# Jan 16^th^ – 18^th^ 2009 (see Appendix 2)
