# Supplementary material for: The Effectiveness of a ‘Train the Trainer’ Model of Resuscitation Education for Rural Peripheral Hospital Doctors in Sri Lanka
Source: PLoS One. 2013 Nov 8;8(11):e79491. doi: 10.1371/journal.pone.0079491 (PMC3821851; doi:10.1371/journal.pone.0079491)
Supplement: Appendix S2 — Instructor workshop (‘training the trainers’) – course outline. (DOCX) [file pone.0079491.s002.docx]

# “Instructor workshop” (Jan 16^th^ – 18^th^ 2009)

### Friday 16^th^ Jan 2009 – Introduction/Orientation

19:00 – Introduction of Participants to Faculty

19:15 - Objectives of Instructor course
 Agenda for 2 days

20:00 - Feedback from last 2 courses – group discussion

20:30 - Dinner faculty and participants

### Saturday 17^th^ Jan 2009 (Day 1)

(SG) Small Groups - 3 to 4 instructor candidates per Consultant trainer

(EG) Entire Group – 16 instructor candidates

8:00 **Lecture** : Becoming a Trainer - Teaching strategy – Dr Mudiyanse Rasnayaka (EG - 30mins) (Consultant Paediatrician)

**Lecture:** Important aspects of setting up a scenario – Dr Mabel Vasnaik (Consultant Anaesthetist)

8:30 **Demonstration** of “Skills station 1-Basic airway management and initial response to an unresponsive patient”. Demonstration of how to teach this skills station to instructor candidates

Group discussion – Questions and Answers about skills station and module from the entire group

8:45 **Practice of “Skills station 1”**. Instructor candidates practice teaching Pre-interns participants under observation of Consultant Instructors, who provide guidance as necessary.

9:45 **Demonstration** of Skills station 2 – Advanced Airway Management (intubation)

Questions and Answers

10:00 **Practice of “Skills station 2”**

11:00 **Morning Tea**

11:30 **Demonstration** of Skills station 3 – Ventricular Tachycardia and Ventricular Fibrillation scenarios

Question and Answers

11:45 Practice of Skills station 3

12:45 **Lunch**

13:45 Demonstration of Skills station 4 – Pulseless Electrical Activity & Asystole Scenarios

Questions and Answers

14:00 Practice of Skills station 4

15:00 **Afternoon Tea**

16:00 Group Discussion – Feed back of Demonstration Participants to the Instructor Candidates and consultant Trainers

Free Practice in Skills stations most in need for improvement

17:00 Close

### Sunday 18^th^ Jan 2009 – Day 2

8:00 **Demonstration** of Skills station 5 – Tachycardia and Bradycardia Algorithms

How to use the Resuscitation Mannequin – Rhythm Generator
 Questions and Answers

8:15 **Practice of “Skills station 5”**

9:15 **Demonstration** of Skills station 6 – Post Resuscitation care and Transport

Questions and Answers

9:30 **Practice** of Skills station 6

10:30 Morning Tea

***Practice workshop with volunteer participants (11:00 to 3:30)***

Consultant Instructors fill out checklist for Instructor candidates as they teach. Feedback and encouragement also given

Each Instructor Candidate is assigned a skills station to teach (because there are 4 per group some will teach two skills stations)

11:00 Show DVD to Participants

- - - Overview of resuscitation
    - Airway management
    - Intubation video (NEJM)

Answer any questions from the Demonstration Participants

11:30 “Instructor Candidates” teach skills station 1 and 2 to Demonstration Participants

12:30 Show DVD to Participants

- - - ALS Algorithm
    - Tachycardia/ Bradycardia
    - Post Resuscitation care & Intubation

13:00 **Lunch**

14:00 Instructor Candidates teach station 2-6 to Demonstration Participants

15:30 **Afternoon Tea**

15:45 De-brief and group feedback from Demonstration Participants. Individual feedback from Consultant instructors

16:30 Session Close – plans for Phase 2
