# Supplementary material for: The Effectiveness of a ‘Train the Trainer’ Model of Resuscitation Education for Rural Peripheral Hospital Doctors in Sri Lanka
Source: PLoS One. 2013 Nov 8;8(11):e79491. doi: 10.1371/journal.pone.0079491 (PMC3821851; doi:10.1371/journal.pone.0079491)
Supplement: Appendix S3 — Resuscitation Training “Instructor Manual”. (DOC) [file pone.0079491.s003.doc]

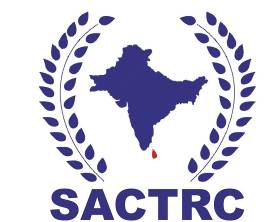

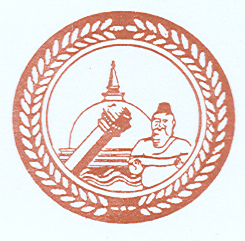


Resuscitation Training

“Instructor Manual”

A collaboration between SACTRC and the Provincial Director of Health Services NCP

# Contents

Resuscitation Training Instructor Manual [2](#__RefHeading___Toc93629828)

Instructor Course Agenda [6](#__RefHeading___Toc93629834)

Adult Learning Behaviour Material [18](#__RefHeading___Toc93629846)

Appendix A : Skills station Guides [28](#__RefHeading___Toc93629847)

Appendix B : Competence checklists [46](#__RefHeading___Toc93629849)

Appendix C : pre-test MCQs [51](#__RefHeading___Toc93629850)

# Resuscitation Training Instructor Manual

### How to use this booklet

This booklet has been designed to aid the instructor workshops with details of the course agenda (subject to change), as well as a resource for Trainers during and after their instructor workshop.

The sections of the booklet are instructor course agenda, adult learning behavior, skills station descriptions, competence checklists and sample pre-test MCQ’s

### Goals of the instructor course

The focus of the course is on “how to teach”. The final goal is to create a trainer who is able to deliver and promote the resuscitation module’s objectives :-

- **Recognition** of a critically ill patient – and calling for help
- **Use of a** **system** for assessing a critically ill patient - **ABCDE**
- **Importance of Basic** life saving maneuvers
- **Update of Advanced** **life support** **algorithms** for the arrested patient
  - **The Nov 2005 guidelines (ILCOR)**
- **“Communication” in Resuscitation**
  - Calling for senior support early
  - Communication and leadership during an arrest
  - Post arrest communication with family and other staff
- **Post Resuscitation care & Inter-hospital Transfer**
- **Practical “Hands on” training**
  - Simulation in real life scenarios using CPR mannequins
  - Assessment with constructive feedback

# Instructor Course

### Residential Training

This will be a two day residential training starting with an inauguration session. At the inauguration session we will deliver the objectives of the Instructor Workshop and explain the focus of this workshop, which is to train the participants **“how to teach”** and how to **“become trainers”**. The dinner is also an opportunity for faculty to meet the participants, which will help facilitate discussion and feedback during the ensuing training sessions.

### The Training Module

The training module that we are teaching the “trainers” how to teach is called the “Peripheral Hospital Training Module” which is a module covering advanced life support which has been accredited by the PGIM and also similar content of the National Training module for Sri Lanka.

This module makes use of a DVD that contains lecture material, and resuscitation mannequins that will be used to carry out training from at total of 6 skills stations to cover the practical aspects of the necessary training in advanced life support.

It was designed to be used by non-specialist staff who are trained to instruct others in resuscitation, hence the use of pre-recorded lectures in the DVD format.

A pilot study showed a positive shift in practice after the deliver of a similar module to participants of a similar resuscitation course.

### Structure of the “Instructor Course”

This course will start with a limited number of presentations on adult learning behaviour, and follow on with a detailed training in how to deliver each of the 6 skills stations which make up the bulk of the “Peripheral Hospital Training Module”. Finally on the second day the trainers will deliver the entire Module to peripheral hospital doctors from the NCP under supervision of the Consultant Instructors. This will be the same module that they are expected to teach on their own at peripheral hospital training rooms starting in February 2009,

### Skills Station Training

Each skill station will be individually demonstrated to the entrire goup by Faculty and discussed to make the specific key learning points clear to the trainers.

The trainers will be divided into groups of 4

For each group there will be:

- 2 consultant instructors,
- 1 mannequin with auxiliary equipment
- 1 Skills station instructor guide
- 4 Simulated course participants (Pre-intern Doctors)

The role of the consultant instructor will be to facilitate each of the 4 Trainers to train take the Pre interns through the skills station in the manner it was shown to them in the entire group.

The consultant instructors is watching to make sure the “Trainer” is able to:-

- Demonstrate the learning contents of the skills station properly
- Engage the participants in Simulation and Scenario based learning
- Assign roles to participants (eg “Team leader”) effectively
- Give constructive criticism and feedback to participants

The consultant instructor is also expected to make sure that each of the 4 trainers has an opportunity to teach the skills station (at least in part) to the pre-intern participants.

We have assigned two consultant instructors per station with a view to having one primary instructor, who has been ideally trained as an “instructor” in a standardised resuscitation course (eg ACLS, ALS, APLS or EMST) where they will be familiar with this adult learning theory and practice, to give feedback on teaching strengths and weaknesses, and a secondary instructor to give feedback issues pertaining resuscitation.

### Putting it all together – Runing a resuscitation Training session

For each group there will be:

- 2 consultant instructors,
- 1 mannequin with auxillary equipment
- 1 Skills station instructor guide
- 4 peripheral hospital participants

It is assumed that by the time we reach this segment of the instructor course all the trainers will be very familiar with each of the individual skills stations, and would have had practice in teaching each of the skills stations to participants.

This segment is about practicing the synthesis of this knowledge on how to teach the individual skills stations into the delivery of the actual training module, which involves the following skills to be developed:

- Delivery of a DVD lecture and encouragement of questions at the end
- Making sure that all the participants in each skills station get equal opportunity to practice scenarios
- How to handle different levels of ability within the participant group
- Keeping to time and schedule
- Ensuring adequate Breaks

At this stage of the instructor course each trainer should be equipped to deliver all 6 skills station as will be required when they are conducting the training course at the peripheral hospital. However, because there are 4 trainers per group they will only be able to deliver 1 skills station each, and 2 will be able to deliver 2 skills stations.

The consultant trainer will pick a trainer to run the next skills as required. They will have a predetermined list for the group of 4 so that the trainers can be utilised sequentially. The trainers will not be told in advance which station they will be teaching, as all should be able to each all stations.

For this segment of the instructor course the Consultant instructor has the important role of filling out an assessment checklist on each Trainer that will be later evaluated to determine if they have made the adequate standard to go on to be trainers in the Phase III of the train the trainer project, or whether further remediation is necessary.

Bishan Rajapakse

Resuscitation Training Program Coordinator

NCP Health-SACTRC

Ph 0773504475

# Instructor Course Agenda

# Instructor Workshop – Schedule and Roles

It is important for all to understand their different roles for this workshop to be effective. Because we are creating “trainers” I have reserved the term “instructor” for “Faculty” (ie consultants who are doing the teaching at this workshop)

I have divided the instructors into two kinds of Instructors for the purpose of running the skills stations and assigned different roles to each. I have also done this because of the varying backgrounds of our faculty.

At this workshop we have instructors who either have completed at least a 2 day “instructor training course” or those who haven’t completed a training course but have significant experience in the field of resuscitation (secondary instructors). either with the American heart association (AHA) advanced cardiac life support (ACLS) course, or UK or Australian resuscitation council’s advanced life support (ALS). And one instructor with extensive experience in advanced paediatric life support, himself having attended the instructor course (APLS), and who will be delivering the lecture program.

I have divided the instructors into Primary instructors who’s role will be on the “teaching” aspects, and secondary instructors who role will be to make sure the content of the “resuscitation” education is fulfilled when we break into groups of 4 students and 2 instructors.

The aim is to have 8 instructors at the course to teach 16 participants.

All the lectures will be given to the entire group, and all the practicals will be carried out with the class split into four group. Each group will have a primary and secondary instructor.

# List of current faculty

(Those who have not yet confirmed are shown in brackets)

Lecture Program – Mudiyanse Rasnayake / Mabel Vasnaik

Skills station Primary Instructors :-

IP1 – Maybel Vasnaik (ACLS instructor – Bangalore)

IP2 – Donnie Woodyard (ACLS instructor – USA)

IP3 – Shakunthala Murthy (ACLS instructor –Bangalore)

IP4 – Jessica Spedding (ALS instructor – UK)

Skills station Secondary Instructors:-

IS1 – Mudiyanse Rasnayake (APLS instructor – Australia & Sri Lanka)

IS2 – Sushila Ranasinghe (Resuscitation Training – Peradeniya Sri Lanka)

IS3 – Chris Nickson (Resuscitation Training – Perth Australia

IS4 – (to be confirmed)

# Schedule Overview

**Pre Workshop session – Friday 16^th^ Jan Evening)**

Overview lecture – Dr Bishan Rajapake

Lecture on “Why resuscitation Training is important – Dr Mabel Vasnaik

**Day 1 – AM (Saturday 17^th^ Jan)**

Lecture on Lecturing and DVD lecturing

Theory on adult education

Practice of lecturing (dvd lecturing)

Lecture on Skills stations and Scenarios

**Day 1 – PM**

Demonstration on Skills stations and Scenarios

Practice of Skills stations and Scenarios

**Day 1 – evening**

Specific description of skills stations 3-6

**Day 2 - (Sunday 18^th^ Jan) whole day**

***“Practice workshop”***

DVD lecture to participants

Skills station 1-2

Morning Tea

DVD lecture 4-6

Skills station 3-6

Lunch

Participants – post test MCQ

Trainers – get lecture and demonstration on how to run a scenario assessment, mark and give feedback

Post Scenario assessments

Close

# Detailed Schedule (Subject to change)

Pre workshop session – (see above)

Day 1 - AM

8:00 **Lecture** : How to give a lecture (including delivering a DVD lecture )

9:00 **Demonstration** of giving a “DVD based lecture”. Demonstration of how to make use of set, environment, dialog, closure.

Faculty acting as both Trainers and Participants

10:00 **Morning Tea**

10:45 **Practice of “Giving a DVD lecture”**.Trainers’ practice teaching Pre-interns participants under observation of Consultant Instructors, who provide guidance as necessary.

# Layout (*group splits into 4)

Key for table:

PN – Prospective trainer (Participant number)

IP – Primary Instructor

IS – Secondary Instructor

DP – Demonstration Participant (Pre-intern)

Numbers

PN : 1-16

IP : 1-4

IS : 1-4

DP : 1-8

Station “A”

Front table (with screen)

Station “C”

Station “B”

Station “D”

RDHS MAIN AUDITORIUM - ANURADHAPURA

### Practical session – Practice of giving a DVD Lecture

**Instructor roles :**

Primary instructor – guiding the situation

Secondary instructor – filling assessment form on giving a lecture

**Station Equipment :** DVD player, Instructional DVD

|  | **A** | **B** | **C** | **D** |
| --- | --- | --- | --- | --- |
| Trainer (performer) | PN1, 2,3,4 | PN 5, 6,7,8 | PN 9, 10, 11, 12 | PN 13, 14, 15, 16 |
| 1° Instructor (evaluator) | IP 1 | IP 2 | IP 3 | IP 4 |
| 2° Instructor (evaluator) | IS 1 | IS 2 | IS 3 | IS 4 |
| Demonstration participant | DP 1, 2 | DP 3, 4 | DP 5, 6 | DP 7, 8 |

For this practical the primary and secondary instructors will observe the Trainers deliver the DVD lectures to the demonstration participants, and facilitate each of the 4 trainers to do this in their station within the allocated time.

### Schedule (cont.)

11:30 **Lecture:** How to teach resuscitation using “Skills stations and Scenarios”

12:30 **Lunch**

13:30 **Demonstration:** How to teach resuscitation using “Skills stations and Scenarios

Demonstration of “Skills station 1 & 2” by Faculty – both acting as Trainers and participants.

Group discussion afterwards

14:30 **Practice of “Skills station 1 & 2”.** (Group splits into 4)

Trainers’ practice teaching Pre-interns participants under observation of Consultant Instructors, who provide guidance as necessary.

### Practical session – Teaching using skills stations and scenarios

**Instructor roles :**

Primary instructor – guiding the situation

Secondary instructor – filling assessment form on how to teach a skills station

**Station Equipment :** (equip for skills stations 1 & 2) Mannequin, OPA, Suction, ETU, Intubateable Mannequin, Airway head, Laryngoscope, ET Tube, introducer, Syringe,

|  | **A** | **B** | **C** | **D** |
| --- | --- | --- | --- | --- |
| Trainer (performer) | PN 5, 6,7,8 | PN 9, 10, 11, 12 | PN 13, 14, 15, 16 | PN1, 2,3,4 |
| 1° Instructor (evaluator) | IP 1 | IP 2 | IP 3 | IP 4 |
| 2° Instructor (evaluator) | IS 3 | IS 4 | IS 1 | IS 2 |
| Demonstration participant | DP 1, 2 | DP 3, 4 | DP 5, 6 | DP 7, 8 |

For this practical the primary and secondary instructors will observe the Trainers teach the content of skills station 1 & 2 (initial approach to an unresponsive patient not breathing, basic and advanced airway management) to the demonstration participants. They will also facilitate each of the 4 trainers to do this in their station within the allocated time.

15:45 **Afternoon Tea**

16:00 **Demonstration:** How to teach resuscitation using “Skills stations and Scenarios

Demonstration of “Skills station 3 & 4” by Faculty – both acting as Trainers and participants.

Discussion

Free Practice in Skills stations most in need for improvement

17:00 Day 1 - Close

## Saturday 17^th^ Jan 2009 – Evening

19:00 Presentation - “Revision of Training objectives – and Agenda for the next days practice training session” (10 mins)

Feedback from ***Trainers*** to Consultant Instructors on Day 1 proceedings – strengths and weaknesses of sessions (20mins)

Demonstration and Informal Practice – Review of Skills stations 3 & 4 (30mins)

Demonstration of how to teach using the defibrillator and how to use the rhythm generator

Opportunity to Practice

20:30 **Dinner** for faculty and participants

## Sunday 18^th^ Jan 2009 – Practice workshop

12 Peripheral hospital Doctors who have not attended previous workshops are the participants to this Training module. They will be divided into groups of 4. The “trainers” will then teach the module according to the following schedule

Consultant Instructors fill out checklist for prospective trainers as they teach. Feedback and encouragement also given after each skills stations

Each Instructor Candidate is assigned a skills station to teach (because there are 4 per group some will teach two skills stations)

## Day 2 – Practice Resuscitation workshop

*Group is split in to 4 from the beginning*

*There are 12 peripheral hospital doctor participants (PHP 1-12)*

**8:00 – 9:00 DVD lectures (deliver presentations 1-3)**

**Instructor roles :**

Primary instructor – guiding the situation & filling assessment form on giving a lecture

Secondary instructor – filling assessment form on giving a lecture

**Station Equipment :** DVD player, Instructional DVD

|  | **A** | **B** | **C** | **D** |
| --- | --- | --- | --- | --- |
| Trainer (performer) | PN **1**, **2**, 3, 4 | PN **5**, **6**, 7, 8 | PN **9**, **10**, 11, 12 | PN **13**, **14**, 15, 16 |
| 1° Instructor (evaluator) | IP 1 | IP 2 | IP 3 | IP 4 |
| 2° Instructor (evaluator) | IS 1 | IS 2 | IS 3 | IS 4 |
| Peripheral Hospital doctor | PHP 1, 2, 3 | PHP 4, 5, 6 | PHP 7, 8, 9 | PHP 10, 11, 12 |

For this session the primary and secondary instructors will observe and **assess** the Trainers deliver the **DVD lectures 1-3** to the demonstration participants. Only one trainer will be able to be observed in the group and if time permits a second person can be asked to run through the skills with the participant (see bolded PN numbers).

(*Trainer Group rotates anticlockwise by one station, and Secondary instructor rotates clockwise by one station*)

**9:00 - 9:30 Practical 1 (Part 1 - teach skills station 1)**

**Instructor roles :**

Primary instructor – guiding the situation & filling assessment form on running a skills station

Secondary instructor – filling assessment form on running a skills station

**Station Equipment :** Skills station 1 (initial approach & Basic Airway Management)

|  | **A** | **B** | **C** | **D** |
| --- | --- | --- | --- | --- |
| Trainer (performer) | PN 5, **6**, **7**, 8 | PN 9, **10**, **11**, 12 | PN 13, **14**, **15**, 16 | PN 1, **2**, **3**, 4 |
| 1° Instructor (evaluator) | IP 1 | IP 2 | IP 3 | IP 4 |
| 2° Instructor (evaluator) | IS 4 | IS 1 | IS 2 | IS 3 |
| Peripheral Hospital doctor | PHP 1, 2, 3 | PHP 4, 5, 6 | PHP 7, 8, 9 | PHP 10, 11, 12 |

For this session the primary and secondary instructors will observe and **assess** the Trainers deliver the **skills station 1** to the demonstration participants. Only one trainer will be able to be observed in the group and if time a second person can be asked to run through the skills with the participant (see bolded PN numbers).

(*Trainer Group rotates anticlockwise by one station, and Secondary instructor rotates clockwise by one station*)

**9:30 - 10:00 Practical 1 (Part 2 - teach skills station 2)**

**Instructor roles :**

Primary instructor – guiding the situation & filling assessment form on running a skills station

Secondary instructor – filling assessment form on running a skills station

**Station Equipment :** Skills station 1 (initial approach & Basic Airway Management)

|  | **A** | **B** | **C** | **D** |
| --- | --- | --- | --- | --- |
| Trainer (performer) | PN 9, 10, **11**, **12** | PN 13, 14, **15**, **16** | PN 1, 2, **3**, **4** | PN 5, 6, **7**, **8** |
| 1° Instructor (evaluator) | IP 1 | IP 2 | IP 3 | IP 4 |
| 2° Instructor (evaluator) | IS 3 | IS 4 | IS 1 | IS 2 |
| Peripheral Hospital doctor | PHP 1, 2, 3 | PHP 4, 5, 6 | PHP 7, 8, 9 | PHP 10, 11, 12 |

For this session the primary and secondary instructors will observe and **assess** the Trainers deliver the **skills station 2** to the demonstration participants. Only one trainer will be able to be observed in the group and if time a second person can be asked to run through the skills with the participant (see bolded PN numbers).

(*Trainer Group rotates anticlockwise by one station, and Secondary instructor rotates clockwise by one station*)

**10:00 - 10:30 Tea break**

**10:30 – 11:30 DVD Lectures (deliver presentations 4-6)**

**Instructor roles :**

Primary instructor – guiding the situation & filling assessment form on giving a lecture

Secondary instructor – filling assessment form on giving a lecture

**Station Equipment :** DVD player, Instructional DVD

|  | **A** | **B** | **C** | **D** |
| --- | --- | --- | --- | --- |
| Trainer (performer) | PN **13**, 14, 15, **16** | PN **1**, 2, 3, **4** | PN **5**, 6, 7, **8** | PN **9**, 10, 11, **12** |
| 1° Instructor (evaluator) | IP 1 | IP 2 | IP 3 | IP 4 |
| 2° Instructor (evaluator) | IS 2 | IS 3 | IS 4 | IS 1 |
| Peripheral Hospital doctor | PHP 1, 2, 3 | PHP 4, 5, 6 | PHP 7, 8, 9 | PHP 10, 11, 12 |

For this session the primary and secondary instructors will observe and **assess** the Trainers deliver the **DVD lectures 3-6** to the demonstration participants. Only one trainer will be able to be observed in the group and if time permits a second person can be asked to run through the skills with the participant (see bolded PN numbers).

(*Trainer Group rotates anticlockwise by one station, and Secondary instructor rotates clockwise by one station*)

**11:30 – 12:00 Practical 2 (Part 1 teach skills station 3)**

**Instructor roles :**

Primary instructor – guiding the situation & filling assessment form on running a skills station

Secondary instructor – filling assessment form on running a skills station

**Station Equipment :** Skills station 3 (Management of cardiac arrest - shockable algorithm VT/ VF)

|  | **A** | **B** | **C** | **D** |
| --- | --- | --- | --- | --- |
| Trainer (performer) | PN **1**, **2**, 3, 4 | PN **5**, **6**, 7, 8 | PN **9**, **10**, 11, 12 | PN **13**, **14**, 15, 16 |
| 1° Instructor (evaluator) | IP 1 | IP 2 | IP 3 | IP 4 |
| 2° Instructor (evaluator) | IS 1 | IS 2 | IS 3 | IS 4 |
| Peripheral Hospital doctor | PHP 1, 2, 3 | PHP 4, 5, 6 | PHP 7, 8, 9 | PHP 10, 11, 12 |

For this session the primary and secondary instructors will observe and **assess** the Trainers deliver the **skills station 3** to the demonstration participants. Only one trainer will be able to be observed in the group and if time a second person can be asked to run through the skills with the participant (see bolded PN numbers).

(*Trainer Group rotates anticlockwise by one station, and Secondary instructor rotates clockwise by one station*)

**12:00 – 12:30 Practical 2 (Part 2 teach skills station 4)**

**Instructor roles :**

Primary instructor – guiding the situation & filling assessment form on running a skills station

Secondary instructor – filling assessment form on running a skills station

**Station Equipment :** Skills station 4 (Management of cardiac arrest – non-shockable algorithm asystole/ PEA)

|  | **A** | **B** | **C** | **D** |
| --- | --- | --- | --- | --- |
| Trainer (performer) | PN 5, **6**, **7**, 8 | PN 9, **10**, **11**, 12 | PN 13, **14**, **15**, 16 | PN 1, **2**, **3**, 4 |
| 1° Instructor (evaluator) | IP 1 | IP 2 | IP 3 | IP 4 |
| 2° Instructor (evaluator) | IS 4 | IS 1 | IS 2 | IS 3 |
| Peripheral Hospital doctor | PHP 1, 2, 3 | PHP 4, 5, 6 | PHP 7, 8, 9 | PHP 10, 11, 12 |

For this session the primary and secondary instructors will observe and **assess** the Trainers deliver the **skills station 4** to the demonstration participants. Only one trainer will be able to be observed in the group and if time a second person can be asked to run through the skills with the participant (see bolded PN numbers).

(*Trainer Group rotates anticlockwise by one station, and Secondary instructor rotates clockwise by one station*)

**12:30 – 13:00 Practical 2 (Part 3 teach skills station 5)**

**Instructor roles :**

Primary instructor – guiding the situation & filling assessment form on running a skills station

Secondary instructor – filling assessment form on running a skills station

**Station Equipment :** Skills station 5 (Management of Tachyarrhythmia’s and Bradyarrhythmias)

|  | **A** | **B** | **C** | **D** |
| --- | --- | --- | --- | --- |
| Trainer (performer) | PN 9, 10, **11**, 12 | PN 13, 14, **15**, 16 | PN 1, 2, **3**, 4 | PN 5, 6, **7**, 8 |
| 1° Instructor (evaluator) | IP 1 | IP 2 | IP 3 | IP 4 |
| 2° Instructor (evaluator) | IS 3 | IS 4 | IS 1 | IS 2 |
| Peripheral Hospital doctor | PHP 1, 2, 3 | PHP 4, 5, 6 | PHP 7, 8, 9 | PHP 10, 11, 12 |

For this session the primary and secondary instructors will observe and **assess** the Trainers deliver the **skills station 5** to the demonstration participants. Only one trainer will be able to be observed in the group and if time a second person can be asked to run through the skills with the participant (see bolded PN numbers).

(*Trainer Group rotates anticlockwise by one station, and Secondary instructor rotates clockwise by one station*)

**13:00 – 13:30 Practical 2 (Part 4 teach skills station 6)**

**Instructor roles :**

Primary instructor – guiding the situation & filling assessment form on running a skills station

Secondary instructor – filling assessment form on running a skills station

**Station Equipment :** Skills station 6 (Post resuscitation care & Transport)

|  | **A** | **B** | **C** | **D** |
| --- | --- | --- | --- | --- |
| Trainer (performer) | PN **13**, 14, 15, **16** | PN **1**, 2, 3, **4** | PN **5**, 6, 7, **8** | PN **9**, 10, 11, **12** |
| 1° Instructor (evaluator) | IP 1 | IP 2 | IP 3 | IP 4 |
| 2° Instructor (evaluator) | IS 2 | IS 3 | IS 4 | IS 1 |
| Peripheral Hospital doctor | PHP 1, 2, 3 | PHP 4, 5, 6 | PHP 7, 8, 9 | PHP 10, 11, 12 |

For this session the primary and secondary instructors will observe and **assess** the Trainers deliver the **skills station 6** to the demonstration participants. Only one trainer will be able to be observed in the group and if time a second person can be asked to run through the skills with the participant (see bolded PN numbers).

(*Trainer Group rotates anticlockwise by one station, and Secondary instructor rotates clockwise by one station*)

**13:30 - 14:30 Lunch**

**14:30 -15:30 Lecture by Faculty on how do run an assessment**

**(whist participants are carrying out a MCQ Post test assessment in another room)**

**Lecture :** How to perform assessments

**15:30 - 16:30 Scenario Post test**

**Instructor roles :**

Primary instructor – guiding the situation & filling assessment form

Secondary instructor – filling assessment form on carrying out an assessment

**Station Equipment :** Skills station 6 (Post resuscitation care & Transport)

|  | **A** | **B** | **C** | **D** |
| --- | --- | --- | --- | --- |
| Trainer (performer) | PN **13**, 14, 15, **16** | PN **1**, 2, 3, **4** | PN **5**, 6, 7, **8** | PN **9**, 10, 11, **12** |
| 1° Instructor (evaluator) | IP 1 | IP 2 | IP 3 | IP 4 |
| 2° Instructor (evaluator) | IS 2 | IS 3 | IS 4 | IS 1 |
| Peripheral Hospital doctor | PHP 1, 2, 3 | PHP 4, 5, 6 | PHP 7, 8, 9 | PHP 10, 11, 12 |

For this session the primary and secondary instructors will observe and **assess** the Trainers get the participants to work take part in a “mega-code” and mark and give appropriate feedback. Each trainer will be given a chance to be primary examiner for one participant, and the others will watch an provide feedback

**16:30 Session Close**

# Adult Learning Behaviour

Becoming a Trainer

# Dr Rasnayaka M Mudiyanse, Senior Lecturer in Paediatrics

Department of Paediatric, Faculty of Medicine, University of Peradeniya, Sri Lanka

This chapter is prepared by summarizing and extracting essential information from the ‘Pocket Guide to Teaching for Medical Instructors’ of the BMJ group.

Teaching and learning

Understand the Learners

Qualities of a Good Trainer

Basic Principals of Teaching

# Teaching and Learning

Teaching is defined as planned experience which brings about a desired change in behavior. Learning is defined as ‘a relative permanent change in an individual’s behavior resulting from experience’. In real life we gather experience and continue to learn. However in teaching, experience is provided in an organized manner with a greater efficiency. Learning experience can be pleasant or unpleasant. Even though we learn from both experiences, tendency with unpleasant experience is to avoid or defense response. Neither of these responses is helpful for achieving teaching goals and therefore should be avoided. Negative remarks, shaming, condemning and even finding and highlighting errors can be consider as unpleasant experience. In practice teachers tend to point out what is wrong even though it may constitute only a small part of the performance. Therefore appreciation of correct performance would be essential for effective teaching. This will be discussed further at the section on giving feedback.

The domains of learning includes

Knowledge

Skills

Attitudes ( being considerate, responsible, arrogant, patronizing)

Relationships ( being able to communicate, delegate, lead)

All four component of learning would be essential for effective performance.

# Knowledge

Knowledge is acquired in six stages

Knowledge – collection of facts

Comprehension – Use the knowledge in different situations

Application – Learner relies upend his/her knowledge to do it in real life of simulated situation

Analysis – Learner attempt to observe in the light of his/her prior knowledge

Synthesis - Analysis leads to a new level of understanding

Evaluation – whole process and the outcome is reflected and judge leading to next course of action

# Skills

There are four stages involved

Perception – learner become aware of the skill and its steps. ( skill training stage 1 & 2 )

Guided response – Learner is actively involved ( skill training stage 3 & 4)

Mastery – able to perform the skill competently. This is achieved by practice

Autonomy – Further practice will achieve a stage when the skill is performed with little conscious thoughts

# Attitudes

The learning of attitudes involves four stages

Perceiving – attitudes like safety, teamwork, sensitivity should be introduced at the beginning of the course.

Complying – usually learners will comply whether they agree or not

Accepting - Achieved by consistent teaching, role modeling and subsequent testing.

Internalization – Reservations are dropped and attitudes become part of the learner

# Relationships

In many situations actual interventions are carried out by team effort. Therefore relationships need special attention in training workshops.

Decisions – Friendly or unfriendly

Tension – Agreeing/disagreeing

Reintegration – Providing support

Communication – Asking for and giving information

Control – Asking for and giving opinion

Evaluation – Asking for and giving guidance

All these features are present in a creative group. However the key to success is depend on the ability to resolve tensions and disagreements. This requires a variety of roles in a training course; time keeper, organizer and a team leader.

# Understand the learner

In an adult education course like NLS or APLS student that we have to entertain different students with diverse knowledge, experience, skills, attitudes, expectation, motivation and ability. Therefore becoming a teacher in a NLS or APLS course would be a new challenge even for an experience teacher. Students in NLS or APLS course are expecting respect for their experience and knowledge.

Teachers should adopt a supportive attitude towards students, especially when they have to play a role of mentor. The needs of the student should be addressed. Maslow’s hierarchy of needs describe the needs of a students.

Physiological needs – sleep, rest, food

Security needs – does he feel confident in expressing his opinion.

Belonging needs – Does the candidate feel accepted by the group and teachers.

Esteem needs – Confidence about own abilities and competence

Cognitive needs – Understand the content and mastering the concepts

Self actualization – Is the students potentials maximized. Does the candidate feel that he is progressing?

Adult learns best when

The content is relevant and has meaning and purpose – at introduction

Learner is actively involved – interactive lectures

Objectives are defined and goals are set – by negotiation with leaner

Positive feedback is given – recognize good thing and things need improvements and how to improve also

Reflection on learning experience is encouraged – though summaries

Even though most of the adult learners are motivated and enthusiastic, you may come across some difficult learners. They can be classified as talker’s, non talkers and destroyers.

**Enthusiastic talkers** can be made use of by giving them a turn to summarize the points discussed. If the discussion is deviated, micro summaries are useful to recapitulate and redirect the discussion. Some time talkers can get in to satellite discussions within the group. Teacher may have to stop the main discussion and listen to the satellite discussion and link it with main discussion. Alternatively teacher may call the particular talker by name to get them back in to the discussion.

**Non talkers** are not equal to non learners. Nervous student need support and encouragement. Teacher should avoid negative, derogatory or destructive responses. If the student fails to answer a question, redirection or rephrasing the initial question may help to avoid embarrassment in front of peers. Teacher may control the situation for example “Sorry I did not phrase the question well”. Repeating and summarizing the candidates ideas may make them feel more comfortable. Questions related his field of work should be given to a nervous candidate as he/she will be more comfortable to answer them.

Third category; **destroyers** can be extremely destructive to learning. They are rare. They consider themselves as having adequate knowledge but fail to share with others, and resist changing or accepting the opinion of the group. Occasionally candidates may be argumentative or rude. This type of learners should be dealt with individually by a senior teacher in private.

# Qualities of a Good Trainer

Teacher should be a **role model**. During a training course their behavior is observed and followed by students. . Appreciation is more effective way of enhancing learning and changing attitudes.

**Asking questions**

Asking questions can facilitate teaching by inducing interactions, allowing student to reflect on their own experiences and providing a feed back to the teacher. However ambiguous and confusing questions, not allowing students to answer or ignoring their response can have negative impact on teaching. Questions should be planned in advance. Intension should not be highlighting lack of knowledge or failures.

Lower level of questions can lead to single word answers whereas higher level of questions can lead to a discussion or debate. Too low level questions can be patronizing and too higher level questions can be threatening. Lower level questions are useful in lectures when quick repetition of data is required. Questions can be phrased to address the deferent stages of knowledge.

**Knowledge** – Knowing is tested by asking to name, list, describe, write, what, when, how, how many.

**Comprehension** – Compare, distinguish, show, try to prove, interpret, and explain

**Application/analysis** – What conclusions, what are the consequences, how could you

**Synthesis** – Design, create, solve this, what do you suggest

**Evaluate** – Evaluate this set of data, according your opinion.., What do you think..

**Positive feedback**

Objective of teaching is to create confidant and capable student. Therefore, giving feedback should be carefully planned to achieve the objective. Natural tendency of a teacher is to observe failures/ inadequacies and to give a feedback considering only the wrong part, ignoring the correct part which may be a major portion of the performance.

This cultural inclination of highlighting inadequacies is not helping for effective teaching and could be overcome by following scheme outlined below.

Ask the learner to tell what went well and what part he is pleased with his performance. This helps to develop self reliance and confidence. Teacher should insist to identify good things. Everybody has something good about them and they should realize it.

Encourage the group to tell what went well and good things about the performance. Drifting to point out failures should be discouraged at this stage.

The teacher/trainer should tell good things about the performance

Ask the learner what points and how he wish to improve if he/she has repeat the performance

Ask the group to suggest points to improve and how to improve them. At this stage also listing what is wrong is not acceptable and trainer should insist on suggestions for improvements.

Teacher also suggests points to improve and how to improve

At the end of the session summarize good points and suggestions for improvements

This whole process is called **positive feedback**. This process has helped the learner to identify his/her strengths and capabilities and where improvements are needed and how to achieve them. Highlighting where went wrong without teaching how to improve becomes a negative feedback and will not help in learning process.

Supporting attitude is very important

# Basic Principals of Teaching

Basic principles of teaching are applicable in lectures, skill teaching, scenario teaching or in conducting discussion groups. They includes

Environment

Set

Dialogue

Closure

Environment – This includes space, seating arrangement, lighting, noise, ventilation, temperature, humidity and any other movements that may disturb or facilitate teaching/learning. Quality of equipments (manikins or audiovisual s) is also very important. Giving attention to environment, organizing them in advance is a responsibility of the instructor and it is essential for an effective teaching session.

Set – Mood of the learners will be set by the environment as well as by explaining the objectives, value and usefulness of the session at the introduction. Explaining the expected role of the student ( Eg - Whether they should interact or carefully listen) during the teaching session is useful. It is important to explain the peculiarities of using manikins in teaching skills and case scenarios. Manikins will not show any changes or responses to indicate progress or improvement of a disease condition. Therefore during skill training or case scenarios the student will have to depend on the information given by the instructor regarding changes of the condition of parameters. However teacher may give false information in order to facilitate next step of planned training. For example when the student has achieved good chest expansion teacher may say the there was no chest expansion. This situation should be explained to the student in advance to avoid confusion.

Dialogue – Actual teaching take place during dialogue. The content should be clear and precise to cover the essentials for the course and delivered within the allocated time. Interactions with the audience to keep the interest and engagement of student also important. For good quality dialogue practice by the teacher is essential.

Closure – Closure includes the time for questioning followed by summery and directing the group for next session. Objective of the summery should be to emphasis the key messages.

## Lectures

Environment – special attention for audiovisual equipments, noise disturbances and quality of slides.

Set – Good environment can set the mood. Interest and enthusiasm can be created by giving an introduction highlighting the importance of the topic and presenting the lay out the lecture. Asking a question to reflect the experience of students can create enthusiasm.

Dialogue – This is the body of the lecture. The content should be complete, precise, clear and relevant to the course. Audiovisual aids such as computer projectors, over head projectors, and flipcharts can be used. Content of a text slides should be limited to 3-5 points. Maximum is 7 line and 7 words in each line. Heading should be limited to 5 words. Picture slides can be incorporated to highlight important points relevant to the topic. However it should not distract the audience from the topic. During the lecture questions can be directed to facilitate interaction. Questions should be clear and precise and should allow any body to answer. Taking answers positively will encourage others to participate in the discussion. Audience activities such as brain storming and buzz groups are useful to create enthusiasm and participation. Brainstorming is when a group is encouraged to express their opinion on a specific question/issue and the opinion is written down without any form of feedback. At the end the lecturer should be able to demonstrate a pattern. Buzz group; small groups within a group is asked to discuss a specific subject in middle of lecture for few minutes. At the end they are asked to present what they have discussed. This method is useful to create energy and enthusiasm. A demonstration at the end or in the middle of a lecture is a useful component in practical courses.

Closure – Closure include question time followed by a summery. It is best to keep the summery to the end so that you will be able to give conclusions and the take home message.

## Skill Stations

**Environment** – All the equipments should be available, functioning and familiar to the teacher. Instructor should check all the equipments before starting the teaching session.

**Set** – By explaining the usefulness and the expected role of the student. Goals of the session should be explained. Four stages of skill training should be explained.

**Dialogue** – This includes four stage skill training.

Stage one; Silent demonstration by the teacher – This is done at the speed of a real situation. It is important to avoid talking as students tend to look at the teachers moth when the teacher is talking rather than steps of actual skill.

Stage two; Demonstration with a commentary. At this stage teacher will explain the skill while performing it. Skill is broken in to several steps and explained. Students are encouraged to ask questions and clarify doubts.

Stage three; Teacher performs the skill while the student is giving the commentary. At this stage teacher can confirm that the student has understood the procedure. Further questioning is encouraged.

Stage four student will perform the skill under supervision and positive critique by the teacher

Stage five; Practice- This may take place after the formal teaching session.

Usually the group is divided in to two at stage three and four to ensure adequate time for all the students.

Closure – Entire group is brought together and allow time for questions and summarizes the important points in the skill and stress the importance of practice. Practice help to master the skill and achieve autonomy when skills are performed almost subconsciously.

## Case Scenarios

**Case scenario** is a form of **improvised role play** in which the management of case is practiced using a manikin and suitable equipments. Student is expected to make decisions according to the guided responses from the instructor. In role play and case scenario participants are expected to act out roles that represent in real life. It helps to teach situations more closely to real clinical situations in life and helps to put knowledge, skills, attitudes and relationships together.

**Teaching scenarios** help to simulate situations closer to real life. Team work can be practiced and experienced teacher could reproduce an almost realistic challenges.

Environment – Environment, equipments and layout should be as realistic as possible. Improvisations should be minimized. Manikins allow procedures to practice. However as they can’t give a physiological feedback student have to depend on the instructor for assessment of the condition of the patient or response to treatment. This situation helps teacher to guide the students and create learning points. When real patients or actors are used as models they should be made up and trained to simulate the condition. However procedures can’t be practiced.

Set – the group should be briefed together and the performing students should be given separate briefing. It is important to ensure that the student who is going to perform clinical intervention has understood the scenario by asking him to repeat the description before he starts the scenario. Peculiarities of using manikins and responses of the instructor should be explained at the beginning.

Dialogue- teacher retain the control by passive responses ( giving response on learners request) or by active responses ( Teacher initiated responses). By these responses teacher retain the control of the direction and outcome of the case.

Closure – Closure should include debriefing when the participant is allowed to express how he felt during and after role play in scenario. Evaluation about the performance with the positive feedback recognizing good things and strengths and pointing out areas to improve and how to improve as explained under the topic ‘ giving feedback ‘ will help to enhance the learning experience. Question time and summery are also essential components for closure in a case scenario.

## Assessment

Instructors assess students formally when the student knows that he/she is been assessed or informally when the student is unaware that he/she is assessed. Formal assessment can be done by using a checklist of score sheet (see appendix).

Assessment can be of two types; formative and summative. When the assessment is done during the course and inform the student the areas to improve it is called formative assessment. This is done in a formal way at the initial pre-course assessment or informally during the course. Summative assessment is done by a final test. Usually knowledge is tested by MCQ and knowledge psychomotor skills and behavior Is tested by case scenario.

# Appendix A : Skills station Guides

**Airway Management Skill Station (1 instructor, upto 6 students - approximately 30 minutes)**

**Key teaching objectives**

By the end of this session, the participant will:

Show competency and confidence in the principles of establishing and maintaining a patent airway and providing adequate ventilation in an airway training manikin

**Equipment at station**

ResusciAnne Skillreporter mannequin

Oropharyngeal Airway, Suction, Oxygen

Ambu mask and bag

**Instructor Information**

The instructor should:

Assess the ability of all participants to maintain an airway and achieve effective ventilation of a manikin measured against predetermined competencies

Allow participants the opportunity to discuss and / or practice airway management and ventilation techniques using an endotracheal tube

”Discuss” the equipment and techniques used for endotracheal intubation including the use of cricoid pressure

This skill station is divided into 2 sections; the first section is the most important.

**1.** All participants **must** receive practical instruction and undergo

assessment of their competency in the following:

• Basic airway opening manoeuvres

- Head tilt / chin lift

- Jaw thrust

• Insertion of airway adjuncts

- Oropharyngeal airway

- Nasopharyngeal airway

• Use of suction

- With expired air +/- supplementary oxygen

• Ventilation using a self-inflating bag-mask with supplementary oxygen

and reservoir

- One person technique

- Two person technique

- Review of ratio of compression to ventilation ratio **pre intubation** followed by a rate of approximately 10 ventilations/minute (with no pause between compressions for ventilations) **post intubation**

**2.** All participants should be offered practical

instruction in, and have the opportunity to practice the skills of advanced airway management and ventilation including:

• **Endotracheal intubation**

• **Ventilation** via the endotracheal tube using a self-inflating bag with supplementary oxygen should be demonstrated.

**Instructor Information**

Set the mood, establish usefulness and state learning objectives for the session.

One method of teaching that you may choose to use is the four-stage approach of:

**Stage 1:** Instructor demonstrates the skill, at normal speed, without explanation

**Stage 2:** Instructor demonstrates the skill more slowly, with explanation

**Stage 3:** Instructor demonstrates the skill while a participant provides explanation

**Stage 4:** Participant demonstrates the skill, with explanation

The practical skills of airway management and ventilation should be taught around a simple scenario. In order not to confuse the participants, this is best achieved by simulating a patient with a respiratory arrest, thereby eliminating the need for chest compressions. The scenario can be broken down into 4 elements:

**Initial approach**

Airway assessment

Basic airway opening manoeuvres

Ventilation with ambu bag

Confirmation of ventilation

**Arrival of basic adjuncts**

Use of suction

Sizing and insertion of oro- and nasopharyngeal airway

Confirmation of ventilation

**Arrival of additional equipment**

Assembly of self-inflating bag-mask, reservoir and supplementary oxygen

Ventilation using one and two person techniques

Confirmation of ventilation

If time permits, allow participants to practice their skills. Also allow participants to ask questions and reflect on the session content before terminating the session with a succinct review of all the major points covered.

**Skills station 2 – Advanced airway management – intubation (1 instructor, upto 6 students - 30 minutes)**

**Key teaching objectives**

By the end of this session, the participant will:

Show competency and confidence in intubating an airway training manikin and coordinating colleagues to support this procedure before during and after the intubation including the application of appropriate cricoid pressure

**Equipment at station**

ResusciAnne Skillreporter mannequin, Airway Trainer (intubating mannequin)

Oropharyngeal Airway, Suction, Oxygen

Ambu mask and bag, Laryngoscope, ET Tube, Introducer, Stethescope

**Instructor Information**

The instructor should:

Assess the ability of all participants to intubate a ventilation of a manikin measured against predetermined competencies

Allow participants the opportunity to discuss and / or practice airway management and ventilation techniques using an endotracheal tube

”Discuss” the equipment and techniques used for endotracheal intubation including the use of cricoid pressure

This skill station is divided into 2 sections – Preparation, and Intubation

**1.** All participants **must** receive practical instruction and undergo

assessment of their competency in the following:

**1. Preparation for intubation (10 min discourse + interactive discussion)**

a) Decision to intubate

- failure to ventilate spontaneously
- Unprotected or threatned airway
  - Secretions
  - Low level of consciousness for whatever cause (usually GCS < 8 as a guide)

b) Preparation for intubation – explanation of necessary conditions:

1) Checking that all essential equipment necessary for intubation in an ideal situation is there ans present in **working** order. (Can use acronym – PC MALES)

- **P** ulse Oximetry
- **C** apnography
- **M** ask (ie. ambu mask & bag –valve checked in skills station 1) - **ESSENTIAL**
- **A** irwary (Oropharyngeal –correct size explained in skills station 1) - **ESSENTIAL**
- **L** aryngoscope (check working light bulb) - **ESSENTIAL**
- **E** ndotracheal Tube (check size and that cuff has no leak) - **ESSENTIAL**
- **S** uction (Yankers suction – check suction working) - **ESSENTIAL**

2) Checking there is adequate staff to carry out intubation. Intubator has to assign these roles to others in the group so all understand what they are doing

- 1 - Intubator

- 2 - Assistant to hand ET tube

- 3- Person to apply cricoid pressure

**2. Endotracheal Intubation (5 min demonstration – 15 mins practice)**

a) Variations on holding Larygnoscope

b) Assigning roles to team members before starting (see above)

c) Correct positioning – Head extension & slight neck flexion (“sniffing the morning air”)

d) Instructing to “start cricoid pressure now”

e) Suction of oropharynx

f) Insertion of larygnoscope and pushing the tongue to the left side and lifting up – not using Laryngoscope as a “lever” against the upper lip or teeth.

g) Communication to the team of either “good view of cords” or “cannot see the cords” and explanation of whether they are either “going to intubate” or “go back to Ambu ventilation”

h) Insertion of tube to correct depth – approx 22cm at the lip in an adult male (Vocal cords sit between two black lines)

i) Inflation of cuff and ventilation via the ET tube using an Ambu bag

j) confirmation of correct tube placement and instruction to “release cricoid pressure”

*NB –if difficulty with intubating within 30 seconds the participant* ***must*** *go back to the ambu ventilation to decrease unnecessary hypoxia (and communicate this to other team members), followed by a further attempt*

3. Confirmation of correct positioning of tube and post intubation care

a) ET intubation

1 – direct visualization for Tracheal intubation (ET tube going through cords)

2 – Chest rising with ventilation & Ascultation – equals BS in 4 places (both axillae

**Instructor Information**

The first section on preparation for intubation should be a basic overview and interactive discussion. The practical skills surrounding the actual skill of intbation should be practiced around a a simple scenario of a patient with a respiratory arrest to get participants in the mind set of working under emergency conditions.

Eg “Mr De Silva is a 58 year old man who has arrived with a low level of consciousness and is not breathing spontaneously. He still has a pulse. We are now going to place a definitive airway”

The fours stage learning model can be used:-

**Stage 1:** Instructor demonstrates the skill, at normal speed, without explanation

**Stage 2:** Instructor demonstrates the skill more slowly, with explanation

**Stage 3:** Instructor demonstrates the skill while a participant provides explanation

**Stage 4:** Participant demonstrates the skill, with explanation

Allow participants to practice their skills rotating through the different roles of intubation eg Intubator, assistant, cricoid pressure, so that participants in the group who are not actively intubating can also be doing something productive such as being a part of the intubating Team. Also allow participants to ask questions and reflect on the session content before terminating the session with a succinct review of all the major points covered.

**Initial Resuscitation/ PEA / Asystole Skills Station (1 instructor, upto 6 students, approximately 30 minutes)**

**Key teaching objectives**

By the end of this session, the participant will be able to:

• Assess the collapsed patient, using an **A B C D E** approach

• Perform effective cardiac compressions & ventilations

• Practice the algorithm for PEA/Asystole including Atropine administration

• Recognise the need for addressing the reversible causes of cardiac arrest

**Equipment at station**

ResusciAnne Skillreporter mannequin

Drug trolley, IV Fluids, Oxygen (intubation equipment optional)

Rhythm generating device or Power point presentation/computer program

**Instructor Information:**

Within this skill station there are three key teaching sections:

a) Initial assessment using an A B C D E approach

b) Correct performance of CPR

c) Recognition of Asystole and PEA and no need for defibrillation

The practical skills of using an A B C D E approach, CPR and defibrillation will be

taught around the scenario described below.

*Clinical case*

"Mr Silva is a 58 year old man who had been complaining of central chest pain 2 hours previously. He has a history of hypertension and is on a “blood pressure pill”. In trying to identify the pill you notice the bottle is empty. His wife tells you that there have been a lot of problems at home lately. On arrival he had a weak pulse of 60bpm, and BP was 90/60. His respiratory rate was 30. When you see him, his is distressed and disorientated".

For ease of teaching, the scenario should be broken down to reflect each section and

the four stage teaching approach must be used.

**Stage 1** Instructor demonstrates the skill, at normal speed, without explanation

**Stage 2** Instructor demonstrates the skill more slowly, with explanation

**Stage 3** Instructor demonstrates the skill while a participant provides

explanation

**Stage 4** participant demonstrates the skill with explanation

A team approach should be used where the instructor is the team leader calling others for help and co-ordinates others to perform a resuscitation. The participants who are not actively involved could critique, and in turn be critiqued when they are being active

**Section A:** **Initial assessment using an A B C D E approach**

*The patient is still conscious but critically ill with rapid breathing and bradycardia and hypotension.*

*The ECG shows bradycardia*

**Instructor information**

The defibrillator is not available during the initial assessment.

Here the instructor just speaks aloud how they would approach this patient in terms of their airway, breathing and circulation.

During participant practice (stage 4), the participants are nominated to take the role of team leader. Here instructors may like to alter the scenario between participants to encourage them to think about their clinical decision-making.

• **Assess responsiveness**

• **Airway** (e.g. patency, debris) and administer high flow oxygen

• **Breathing adequacy** (e.g. **respiratory rate**, expansion, percussion, breath sounds, SpO2 eg.)

• **Circulatory status** (e.g. pulse, blood pressure, capillary refill, urine output, look for evidence of haemorrhage (drains, PR bleeding) – IV fluids, management of bradycardia


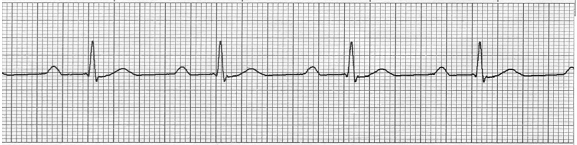
 • **Disability** (**conscious level**) (e.g. AVPU, pupils)

• **Exposure**

**Section B : Correct performance of CPR**

*Clinical case progression*

-The patient becomes unresponsive, and there is NO carotid pulse palpable, but the cardiac monitor still shows a Bradycardia

• Call for **help**

• Open **airway**

• Check **breathing and circulation**

• Call **resuscitation team** and **send for defibrillator**

• Deliver **effective chest compressions**

• Instructs assistant to commence bag-mask ventilation

• Correct ratio **30:2**

• Follow algorithm – ie Atropine, and Adrenaline

**Section C: Recognition of Asytole and the Non-shockable limb of the algorithm**

*Clinical case progression*

-CPR 30:2 is in progress. The defibrillator arrives – nurse alerts doctor that it is here. (Instructor can use different members of the group to role play). They rhythm has now changed to:-

**Instructor information**

Emphasise “team leadership” in running the arrest.

Also emphasise going through the list of “reversible cause of cardiac arrest” and that this applies to both Shockable and Non-shockable rhythms

**1 participant** can take on team leader while **2 participants** should perform the chest compression / ventilation. The other **participants** can help the team leader focus on running the cardiac arrest, but take on other roles such as being the scribe etc. The scenario can be stopped at any point to get comments from the participants who are not running the arrest for “critique”. The different participants should be rotated through all roles during the course of the station.

**Instructor Information – points for discussion / further demonstration**

• Early assessment addressing ABC’s with treatment

potential role of poisong in this case (one of the reversible causes)

Calling the resuscitation team

• The role of the precordial thump (in this case –“No role”)

• Combined assessment of the breathing and circulation

• **Minimise interruption** to chest compressions

• The role of **prevention** of arrest by early resuscitation

- Understanding what PEA is and how there can be electrical activity without a pulse
- Understanding Asytole (and how fine, low amplitude VF is treated as asystole)

Allow participants to ask questions and reflect on the session content before

terminating the session with a succinct summary of all the major points covered.

**Summary**

• Rapid and effective patient assessment

• Provision of effective ventilation and compressions

• Recognition of PEA and Asystole

• Non- Shockable limb of the ALS treatment algorithm

• Theory and practice of safe defibrillation.

**Initial Resuscitation/Defibrilation/Vfb/VT Skills Station (1 instructor, 6 students, approximately 30 minutes)**

**Key teaching objectives**

By the end of this session, the participant will be able to:

• Assess the collapsed patient, using an **A B C D E** approach

• Perform effective cardiac compressions and ventilations

• Recognise the need for defibrillation

• Perform safe defibrillation

**Equipment at station**

ResusciAnne Skillreporter mannequin

Drug trolley, IV Fluids, Oxygen (intubation equipment optional)

Rhythm generating device or Power point presentation/computer program

Defibrillator

**Instructor Information:**

Within this skill station there are three key teaching sections:

a) Initial assessment using an A B C D E approach

b) Correct performance of CPR

c) Recognition of VF and safe defibrillation

The practical skills of using an A B C D E approach, CPR and defibrillation will be

taught around the scenario described below.

*Clinical case*

"Mr Sunil is a 56 year old man with a history of hypertension and Diabetes admitted to the ward with acute severe chest pain. He has been given aspirin, nitroglycerin sublingually and morphine. You are the house officer doing the night shift when you are called to see the patient as he has developed worsening chest pain."

For ease of teaching, the scenario should be broken down to reflect each section and

the four stage teaching approach must be used.

**Stage 1** Instructor demonstrates the skill, at normal speed, without explanation

**Stage 2** Instructor demonstrates the skill more slowly, with explanation

**Stage 3** Instructor demonstrates the skill while a participant provides

explanation

**Stage 4** participant demonstrates the skill with explanation

A team approach should be used where the instructor is the team leader calling others for help and co-ordinates others to perform a resuscitation. The participants who are not actively involved could critique, and in turn be critiqued when they are being active

**Section A:** **Initial assessment using an A B C D E approach**

*The patient is still conscious but critically ill with chest pain*

**Instructor information**

The defibrillator is not available during the initial assessment.

Here the instructor just speaks aloud how they would approach this patient assign their airway, breathing and circulation.


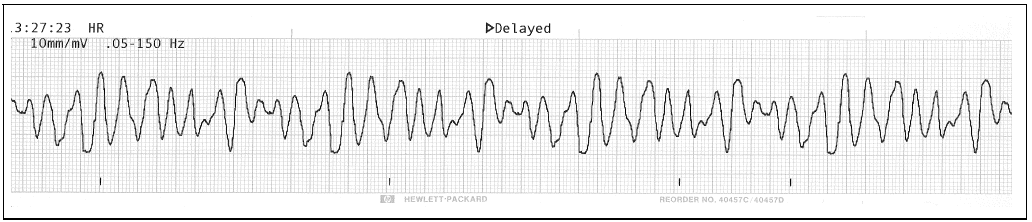
During participant practice (stage 4), the participants are nominated to take the role of team leader. Here instructors may like to alter the scenario between participants to encourage them to think about their clinical decision-making.

• **Assess responsiveness**

• **Airway** (e.g. patency, debris) and administer high flow oxygen

• **Breathing adequacy** (e.g. **respiratory rate**, expansion, percussion, breath sounds,

SpO2)

• **Circulatory status** (e.g. pulse, blood pressure, capillary refill, urine output, look for evidence of haemorrhage (drains, PR bleeding))

• **Disability** (**conscious level**) (e.g. AVPU, pupils)

• **Exposure**

**Section B : Correct performance of CPR**

*Clinical case progression*

-The patient becomes unresponsive

• Call for help

• Open airway

• Check breathing and circulation

• Call resuscitation team and send for defibrillator

• Deliver effective chest compressions

• Instructs assistant to commence bag-mask ventilation

• Correct ratio 30:2

• Continue until monitor/defibrillator attached

**Section C: Recognition of VF and safe defibrillation**

*Clinical case progression*

-CPR 30:2 is in progress. The defibrillator arrives. The following rhythm is seen

**Instructor information**

Emphasise “safety” to all participants and be prepared to intervene to stop the scenario

if necessary.

**2 participants** should perform the chest compression / ventilation during

stages 1 - 3 so that the other **4 participants** can focus on defibrillation. Participants can take over ventilation and chest compression roles during stage 4.

During defibrillation – emphasise the need to minimise delays in chest compression.

• Correctly apply paddles / gel pads (if available), monitoring leads whilst CPR

continues

• Select correct energy level (biphasic / monophasic)

• Stop CPR and identity VF on monitor

• Remove free flowing oxygen

• Warn team to stand clear,

• Charge defibrillator, rapid visual check, confirm VF, deliver shock

• Immediately resume CPR without rechecking rhythm

• Indicate that CPR should be continued for 2 minutes prior to checking rhythm

**Instructor Information – points for discussion / further demonstration**

• Calling the resuscitation team

• The role of the precordial thump

• **Combined assessment** of the breathing and circulation

• **Minimise interruption** to chest compressions

• Transthoracic impedance (effect of gel pads, paddle pressure etc)

• Environmental hazards / risks

• **Biphasic / monophasic** waveforms

• ‘Dumping’ the energy from charged paddles

• **Safety** ( Participants call “stand back”. Charge paddles on patient or in the defibrillator

Don’t wave paddles in the air, caution with oxygen. No need to remove cardiac monitor leads)

Allow participants to ask questions and reflect on the session content before

terminating the session with a succinct summary of all the major points covered.

**Summary**

• Rapid and effective patient assessment

• Provision of effective ventilation and compressions

• Recognition of VF

• Shockable limb of the ALS treatment algorithm

• Theory and practice of safe defibrillation.

**Tachycardia/Cardioversion/Bradycardia Skills station**

Instructor will review how to read a rhythm strip:

1. Is there any electrical activity?

2. What is the ventricular (QRS) rate?

3. Is the QRS width normal or prolonged?

4. Is atrial activity present? (If so what is it? Normal P waves? Other atrial activity?)

5. How is atrial activity related to ventricular activity?

**Tachycardia**

Key teaching objectives

• Recognition of broad complex tachycardia and narrow complex tachycardia

• Principles of treatment, indications for chemical/electrical cardioversion

• Mechanics of safe synchronized cardioversion

Show examples of Supraventricular tachycardia, rapid atrial fibrilation, ventricular fibrillation, ventricular tachycardia asking students to describe whether it is narrow/wide complex, regular or irregular

Review indications for synchronized cardioversion

Explain how to set up for synchronized cardioversion, paddle placement.

Stress importance of not shocking during vulnerable ventricular repolarization phase- "T" wave as this could result in the deadly vfib rhythm

Present 2-3 scenarios and choose a participant to go through each scenario:

25 year old woman with no prior medical history presents with palpitations, and lightheadedness. patient appears anxious.

VS BP 115/85 HR 180 RR 20 O2 saturation 98%

ABC approach

Oxygen, IV access, monitor

Monitor shows SVT.

Student should go through algorithm, first recognizing that this patient is stable with a narrow complex regular tachycardia

Then discuss treatment options: vagal maneuvers (ask participants to give examples: cartodi sinus massage, valsalva,etc)

Vagal maneuvers fail.

Next option: adenosine 6mg IV push (discuss how this medicine has a short half-life on the order of seconds and should be given with a stopcock with a saline flush- so as to facilitate delivery to the heart quickly), may repeat 12mg x2. Another option is verapamil 5mg IV push.

iI Patient breaks, then need 12 lead ekg)

2nd case:

63 year old man with h/o dm, htn, 2 days post op from surgery- noted to have tachycardia. pt, c/o some shest pain and palpitations.

HR 145, BP 80/40 RR22, o2 sat 98%

ABC approach

Oxygen, IV access, monitor

Monitor shows irregularly irregular

Participant should recognize that patient is unstable- needs immediate synchronized cardioversion

If synchronized cardioversion performed. symptoms resolve, hr 110, bp 110/75

now participant can reassess algorithm: now stable with narrow complex irregular tachycarida--> atrial fibrillation

treatment options: rate control: digoxin, b-blocker

<48 hours: consider amiodarone.

3rd case:

65 y.o man with h/o multiple myocardial infarction p/w presyncopal symptoms, palpitations

Hr 170 bp 160/100 rr 20

ABC approach, iv access, o2 monitor

monitor shows ventricular tachycardia

participant recognizes, stable, broad complex, regular tachycardia--> V Tach

Management: amiodarone 300mg IV

**Bradycardia**

Objectives

Recognise bradycardia and differentiate between the different degrees of heart block

• Understand the principles of treating bradycardia

• Understand the indications for cardiac pacing

• Be aware of the different methods available for cardiac pacing

Choose 3 participants (one as a team leader, the other 2 as assistants) guide them through the following scenario. prior to starting review algorithm and discuss what is meant by adverse signs: bp <90, Hr< 40, heart failure, vent arrythmias, also review risk factors for asystole)

Clinical Scenario for Bradycardia simulation

You are called to see a 60 year old patient who has developed complete heart block (CHB) after an acute inferior myocardial infarction.

Clinical Course

• Initially – reduced conscious level.

Airway: clear;

Breathing: RR 14 min-1, fine basal crackles, C: P 40-50 min BP 70/40, CRT 4 sec; D: verbal response; E: nil

• Unresponsive to atropine.

• Patient collapses – Initial rhythm PEA (CHB 40-50 min-1); continue until relevant reversible causes

excluded

• VF followed by return of spontaneous circulation after 2nd shock; P 80 min-1; BP 90/40; starts to

breathe

Interventions – Key treatment points in bold to emphasize

**Initial approach**

Complete heart block

ABCDE approach

Oxygen, IV access

Recognise compromised bradycardia

Atropine 0.5 mg (further increments up to 3 mg)

Request transcutaneous pacing

Cardiac arrest management

PEA Check patient (breathing / circulation)

Call resuscitation team / help

2 min CPR (30:2)

Airway / ventilation / oxygen

Attach ECG monitoring (if not already)

Give first adrenaline 1 mg IV

Recognise and treat relevant reversible causes

(drugs / electrolyte disturbances)

# (further cycle as required)

VF Check monitor / confirm rhythm

1st shock (150-200 J biphasic or 360 J monophasic)

2 min CPR (continuous chest compression / ventilation)

VF Check monitor / confirm rhythm

Give further adrenaline after 3-5 min*

Minimise interruptions in CPR

2nd shock (150-360 J biphasic or 360 J monophasic)

2 min CPR (continuous chest compression / ventilation)

NSR Check monitor / confirm rhythm

Check patient (signs of life / pulse)

Post resuscitation care Comments

**Post Resuscitation Care Skills station**

Instructor will review important elements of post resuscitation care:

Indications for Intubation

Post Intubation Management

Ongoing reassessment and monitoring of patient

Indications for Intubation:

1)Airway Protection Ask yourself can they talk? Can they swallow and manage secretions?

What is the level of consciousness?

GCS <9 indicate “potential” airway compromise due to lack of pharyngeal muscle tone & reflexes

2) Failure to maintain Ventilation/Oxygenation:

Is the SaO2 <90% on High Flow O2 or PaO2<60 on FiO2>40%?

Is the PaCO2 >55 if baseline is normal, or >10 increase from baseline

What is the Respiratory Rate and can this rate be maintained?

3) Expected decline in Clinical Status (Instructor should spend time discussing need for early intubation if you expect that a patient’s status is likely to decline during transport)

Deterioration/Impending Compromise

Transport

Airway protection during procedures (ie. endoscopy)

Postintubation Management

1) Confirm intubation (end tidal CO2, calorimeter, Chest x-ray)

2) Secure tube

3) Post Intubation Medications (sedation, paralysis)

Diazepam 0.2 mg/kg

Lorazepam 4-6 mg

Midazolam 0.1 mg/kg bolus, then 0.1 mg/kg/hr 2-5 mg/hr (Drip 50 mg in 250 cc NS, Start at 10-25 cc/hr)

Propofol .5-1 mg/kg then 25-100 mcg/kg/min, start at 10 cc (100 mg)/hr which correlates with 1 mg/kg/hour

Vecuronium .1 mg/kg then .03 mg/kg q25-45 min or 1-2 mcg/kg/min

4) Patient position: if possible place head of bed at 30 degrees

5) Nasogastric tube

6) ABG

7) Cuff pressure

8) foley catheter

Ongoing assessment and Monitoring

1. all patients after resuscitation should have close monitoring of vital signs, and be placed on a continuous cardiac monitor
2. foley catheter should be placed and urine output should be monitored

3) approach all changes in vital signs in a systematic fashion as described below:

*Bradycardia*

Assume hypoxia and therefore tube displacement until proven otherwise

*Desaturation (mnemonic DOPE)*

**D**isplaced tube

**O**bstruction-pass suction catheter through tube

**P**neumothorax

**E**quipment failure-take off vent and bag patient

if all of the above have been evaluated, consider shunt physiology

Hypotension

· Pneumothorax

· Decreased Venous Return from positive pressure ventilation, disconnect from vent for 30-60 seconds and observe for increased Blood Pressure and decreased heart rate. Consider reducing PEEP and decreasing Tidal Volume. Auto-PEEP in obstructive airway disease.

· Excessive hyperventilation-

· Induction Agents-diagnosis of exclusion, give fluid bolus

· Cardiogenic-fluid bolus

Present scenarios and choose a participant to go through each scenario:

Mr. Chandradasa, a 62 old man, with a history of Hypertension, Diabetes presented with CHF exacerbation subsequently developed PEA underwent resuscitation had return of spontaneous circulation- and now has been intubated. What steps are you going to take to manage this patient post intubation?

Student should discuss post intubation care as outlined above (securing tube, sedation, nasogastric tube) as well as ongoing assessment and monitoring of the patient.

Now present another student with a change in the hemodynamics of the patient-

“A nurse comes to you and says, Doctor, Mr. Chandradasa BP is 70/40 and his Heart rate is 130” Have the student go through the differential of Hypotension (as discussed above) and discuss what they may do to correct the problem.

In this case the patient could be off the vent and receiving bag-mask ventilation but the ventilation is excessive**. Instructor can simulate a Nurse who is bagging the patient excessively** and see if the student notices and instructs the nurse to decrease rate of ventilation in which case the hemodynamics improve.

Now present another student with a desaturation in Mr. Chandradasa. Help them go through the differential of desaturation (DOPE mnemonic) and describe what they would do to rule out items in the differential. (i.e recheck tube placement, in line suction to rule out obstruction, repeat physical exam and/or CXR to look for pneumothorax, and taking the patient off the vent and performing bag mask ventilation to evaluate for equipment failure) Instructor should emphasize to students the importance of taking patient off ventilator in this situation as well as during any need for repeat resuscitation.

### Scenario 2 – Patient Transfer

This scenario is to make sure that doctors do the basics before transferring patients to another Hospital

1. Organise the transfer with receiving hospital – eg Consultant or ICU SHO/MO in General or Teaching hospital. Important to communicate
   1. Name, Age, Sex
   2. Condition of the patient – Especially Airway Status – (eg intubated or not, Bag mask ventilated or not, level of oxygen delivery-prongs mask etc, and GCS)
   3. Reason for Transfer (eg Intubated or no facilities, critical illness)
   4. Plan for transfer (eg With or without medical staff, bag mask ventilated or mechanical ventilation, Fluids running, Catheter or other tubes insitu)
   5. Expected duration of trip and time of arrival in their institution
2. Re asses and monitoring of patient prior to transfer
3. **IV Access** and start fluids if necessary
   1. Enough fluids if necessary & Long Journey and
4. **Oxygen Delivery Plan**
   1. Is Nasal prongs adequate ?
   2. If via mask – is oxygen supply adequate?
   3. Do they need to be intubated?
5. Medical staffing Plan
   1. Is Solo transfer adequate
   2. If no resources for accompanying patient what precautions have been taken
      1. Position patient on side? Airway adjunct?
6. Position of Patient
   1. Supine in intubated or well patient
   2. On side if airway threatened and not intubated
7. DOES THE PATIENT NEED TO BE INTUBATED PRIOR TO TRANSFER
   1. See indication for intubation above
   2. If in doubt **Call the person at the receiving hospital** and Ask for their advice stating:
      1. Patients age & sex, co-morbidites
      2. Airway status (secretions or not), respiratory rate, Saturations, Chest movements, and GCS
8. Have you written a detailed transfer note with all the vital signs at the time of transfer?
   1. Transfer notes are a good way of remembering vital information

Instill in the student that the most important reversible causes of cardio respiratory arrest are HYPOXIA and HYPOVOLAEMIA - this frequently occurs after interhospital transfer which are both preventable by good care and forward planning., and taking precaution

**A good strategy is “PLAN for the WORST situation, and then HOPE for the BEST”**

Mr Tikirbanda is a 58 year old farmer who presented with sudden onset left sided paralysis. He also has slurred speech and he is only responsive to verbal stimulation. His pulse is 60, BP 120/80 and RR 24, and saturations are unavailable.

You suspect a stroke and want to organize a transfer to the General hospital which is 1 hour away.

Whilst dong this the nurse tells you that Mr Tikirbanda’s breathing patter has changed, now more rapid and there is some drooling. He does not respond to your voice, but is easily stimulated by a sternal rub, and localized the pain, and also give you a groan. His eyes are now closed but open with the pain also

***Get participant to act out the scenario:***

Instructor pretends to be the Doctor on the Receiving station and has a conversation with the MO in the language of their choice.

Participant is led through the phone call, and has to demonstrate management of ABCD, and a plan for transfer.

Also go through to the stage of transfer onto a trolley an position of the Mannequin (Patient)

Patient transfer should occur with at lest 3 people with one for the head

Patient should be managed on the side

# Appendix B : Competence checklists

(for instructors to mark “trainers”)

**Trainer Competence Assessment - overall**

This checklist is to be carried out by Consultant Instructors to ensure that the Trainers demonstrate adequate skills in teaching resuscitation. Theses are based on “General observations” of Trainer particularly in the first part of the workshop - Skills Station Training, and “Objective assessment” in the Teaching of a scenario based skills station in the Peripheral Hospital Training Module.

**Consultant Instructor Name: Date:**

**Trainer being Assessed:**  **Setting of observation:** General Observations / Objective Assessment

| Competency | Successful (comments) | Needs Remediation | Plan / advice given |
| --- | --- | --- | --- |
| **Communication -** Communicates ideas and concepts clearly, maintains positive rapport with learners. uses appropriate nonverbal communication skills, uses appropriate terminology for audience |  |  |  |
| **Manage Technology** – Uses technology associated with teaching (such as manikins, rhythm generators and other teaching aids) and uses audio/video technology effectively |  |  |  |
| **Stimulation and Motivation** – Provides a stimulating learning environment that maintains the interest of students. Encourages interaction from participants. |  |  |  |
| **DVD presentation** – able to make sure that participants understand the main points of the DVD presentations |  |  |  |
| **Questioning and Feedback** - Interacts with participants with appropriate questions to evaluate their understanding and thinking process. Gives feedback appropriately, particularly when skill is not being learned |  |  |  |

Lecture – Feedback Form

Name of the lecturer -……………………………Name of the Evaluator-…………..

Topic - ……………………………………………Time starting -------------, End ………

Set and environment

|  | Did not achieved | Achieved | Good |
| --- | --- | --- | --- |
| 1. Check and adjust lay out |  |  |  |
| 2. Check equipments |  |  |  |
| 3. Welcome and introduce self |  |  |  |
| 4. Sets the mood |  |  |  |
| 5. Establishes the usefulness |  |  |  |
| 6. States learning objectives |  |  |  |
| 7. Overall performance of SET |  |  |  |

Dialogue

|  | Did not achieved | Achieved | Good |
| --- | --- | --- | --- |
| 1. Presents material in a clear, logical sequence |  |  |  |
| 2. Uses visual aids appropriately |  |  |  |
| 3. Ensure voice projection |  |  |  |
| 4.Address/involves the audience |  |  |  |
| 5. Uses the eye contact appropriately |  |  |  |
| 6. Demonstrates enthusiasm |  |  |  |
| 7. Uses humour appropriately |  |  |  |
| 8. Uses personal/ audience experience |  |  |  |
| 9. Asks appropriate questions |  |  |  |
| 10. Responds positively to answers |  |  |  |
| 11. Keeps to time |  |  |  |
| 12. Attitude/behavior encourages learning and interactions |  |  |  |
| 13. Overall performance of Dialogue |  |  |  |

Closure

|  | Did not achieved | Achieved | Good |
| --- | --- | --- | --- |
| 1. Invites and answer questions |  |  |  |
| 2. Returns to learning objectives and summarizes |  |  |  |
| 3. Terminate the session |  |  |  |
| 4. Overall performance of closure |  |  |  |

General Comments ( what do you feel if you are a trainee )

|  |
| --- |

Skill Teaching – Feedback Form

Name of the Taecher -……………………………Name of the Evaluator-……………………..

Topic - ……………………………………………Time starting -------------, End ………………

Set and environment

|  | Did not achieved | Achieved | Good |
| --- | --- | --- | --- |
| 1. Check and adjust lay out |  |  |  |
| 2. Check equipments |  |  |  |
| 3. Ensure all the candidates can see |  |  |  |
| 4. Welcome and introduce self |  |  |  |
| 5. Sets the mood |  |  |  |
| 6. Establishes the usefulness |  |  |  |
| 7. Clarifies the role of learners and teacher |  |  |  |
| 8. States learning objectives |  |  |  |
| 9. Overall performance of SET |  |  |  |

Dialogue

|  | Did not achieved | Achieved | Good |
| --- | --- | --- | --- |
| 1. Describe 4 stage method of teaching skills |  |  |  |
| 2. Demonstrate the skill without commentary |  |  |  |
| 3. Demonstrate the skill with commentary |  |  |  |
| 4. Demonstrate the skill with candidate commentary |  |  |  |
| 5. Candidate demonstrate with appropriate commentary |  |  |  |
| 6. Keep check on safety |  |  |  |
| 7. Relate skill to the other aspects of the course |  |  |  |
| 8. Enables practice if time allows |  |  |  |
| 9. Attitudes/behavior encourages learning and interactions |  |  |  |
| 10. Overall performance of Dialogue |  |  |  |

Closure

|  | Did not achieved | Achieved | Good |
| --- | --- | --- | --- |
| 1. Invites and answer questions |  |  |  |
| 2. Returns to learning objectives and summarizes |  |  |  |
| 3. Terminate the session |  |  |  |
| 4. Overall performance of closure |  |  |  |

General Comments

|  |
| --- |

Scenario Assessment – Feedback Form

Name of the Teacher -……………………………Name of the Evaluator-……………………..

Topic - ……………………………………………Time starting -------------, End ………………

Set and environment

|  | Did not achieved | Achieved | Good |
| --- | --- | --- | --- |
| 1. Check and adjust lay out |  |  |  |
| 2. Check equipments |  |  |  |
| 3. Check whether assistant is familiar with scenario and his/her role |  |  |  |
| 4. Welcome and introduce self |  |  |  |
| 5. Sets the mood |  |  |  |
| 6. Establishes the usefulness |  |  |  |
| 7. Clarifies the role of the teacher and learner |  |  |  |
| 6. States learning objectives |  |  |  |
| 7. Overall performance of SET |  |  |  |

Dialogue

|  | Did not achieved | Achieved | Good |
| --- | --- | --- | --- |
| 1. Brief candidate |  |  |  |
| 2. Allows questions |  |  |  |
| 3. Checks understanding |  |  |  |
| 4. interact constructively |  |  |  |
| 5. Keeps check on safety |  |  |  |
| 6.Keeps to time |  |  |  |
| 7. Attitudes and behavior encourage learning and interaction |  |  |  |
| 8. Overall performance of dialogue |  |  |  |

Closure

|  | Did not achieved | Achieved | Good |
| --- | --- | --- | --- |
| 1. Asks the candidates to Waite out side confirm results with the colleague |  |  |  |
| 2. Reaches a correct decision |  |  |  |
| 3. Inform the candidate of the satisfactory result |  |  |  |
| OR |  |  |  |
| 4. Counsel the candidate of the need for reset |  |  |  |
| 8. Terminate the session |  |  |  |
| 9. Overall performance of closure |  |  |  |

General Comments

|  |
| --- |

# Appendix C : pre-test MCQs

**Phase 1b Pretest:**

1) Which of the following statements is FALSE regarding the recognition of the critically ill patient and prevention of cardiorespiratory arrest?

1. airway, breathing and circulation problems can cause cardiorespiratory arrest
2. early recognition and treatment of critically ill patients never prevents cardiorespiratory arrests
3. most patients who have an in-hospital cardiac arrest have warning signs and symptoms before the arrest
4. all critically ill patients should be given oxygen, although you may aim for a lower oxygen saturation in patients with COPD
5. the ABCDE approach can be used to treat critically ill patients

2) You are called to evaluate a patient who is found unresponsive on the ward. Which of the following best shows the correct sequence of actions that you will take in your initial approach to this patient?

1. Call for help, ensure personal safety, open the airway, assess for breathing and check pulse, if no breathing give 2 breaths
2. Call for help, check for responsiveness, if no response start chest compressions, give 2 breaths
3. Tell the nurse that you need to drink some tea and then you will come and assess the patient
4. Ensure personal safety, check for responsiveness, call for help, open the airway, assess for breathing and check pulse, if no breathing give 2 breaths

3) All of the following are true about airway obstruction in an unconscious patient **EXCEPT**:

1. sometimes the obstruction can be relieved by airway opening maneuvers like head tilt, chin lift, jaw thrust
2. airway obstruction can be worsened while inserting an oropharyngeal airway because the tongue may be pushed backwards
3. if you suspect a cervical spine injury, use a jaw thrust or head tilt in combination with manual in-line stabilization to open the airway
4. if possible, give oxygen at high concentration while attempting to relieve airway obstruction
5. sometimes airway obstruction only becomes apparent after starting ambu-bag ventilation

4) Which of the following rhythms (shown in figures 1-4 below) should NOT be shocked in the setting of cardiac arrest in which the patient is pulseless?

1. Figures 1 and 2
2. Figures 1 and 3
3. Figures 2 and 4
4. Figures 1,2, and 3
5. Figure 3 only
6. Figure 2 only
7. Figure 1 only
8. All of the rhythms are shockable
9. None of the rhythms are shockable

Figure 1

Figure 2

Figure 3

Figure 4

5) A patient is found unresponsive on the ward. You confirm unresponsiveness and call for help. After further evaluation, you find that the patient is apneic and pulseless. You begin CPR. A monitor shows ventricular fibrillation. Which of the following best represents the correct sequence of actions you will take to further manage and treat this patient?

1. give 3 shocks pausing briefly between each shock to reassess the rhythm, if ventricular fibrillation persists, resume CPR at a rate of 30:2
2. give 1 shock and then pause to reassess the rhythm on the monitor and feel for a pulse, if ventricular fibrillation persists then resume CPR at a rate of 30:2
3. give 1 shock then immediately resume CPR at a rate of 30:2 for approximately 2 minutes, then pause to reassess the rhythm on the monitor and feel for a pulse
4. give 3 shocks pausing briefly between each shock to reassess the rhythm, and give adrenalin 1mg IV prior to the third shock, then immediately resume CPR at a rate of 30:2 for approximately 2 minutes, then pause to reassess the rhythm on the monitor and feel for a pulse

6) Which of the following statements is **FALSE** regarding resuscitation in the setting of ventricular fibrillation/pulseless ventricular tachycardia?

1. if a defibrillator is not immediately available, a single precordial thump can be give
2. the interval between stopping chest compressions and delivering a shock should be minimize
3. adrenalin is given before the delivery of the 3rd shock and this 3rd shock should be delayed if the adrenalin is not ready to give
4. rhythm checks should be brief and signs of life and pulse checks taken only if there is an organized rhythm on the monitor

7) Which of the following is **NOT** a potential reversible cause of Pulseless electrical activity?

1. Hypokalemia
2. pulmonary embolism
3. hypothermia
4. hypovolemia
5. thyrotoxicosis

8) Which of the following is **TRUE** regarding medications used in the management of cardiac arrest?

1. if intravenous access is difficult to obtain, medications should never be given via the endotracheal tube
2. the dose of amiodarone in the setting of refractory ventricular fibrillation is 150mg
3. sodium bicarbonate should be given in all cardiac arrests to reverse metabolic acidosis
4. the role of medications during cardiac arrest is secondary to effective chest compressions, defibrillation, and effective ventilations
5. atropine can be given in pulseless electrical activity but not asystole

9) A 68 year old woman presents with shortness of breath. The patient is given oxygen and intravenous access is obtained. A monitor shows the following rhythm:

Blood pressure is 70/40 and the patient is diaphoretic. Which of the following is the most appropriate action to take in order to manage this patient?

1. defibrillate at 360 joules (monophasic)
2. adrenalin 1 mg IV
3. call the cardiologist
4. atropine 3mg IV
5. atropine 0.5 mg IV

10) In the tachycardia algorithm, all of the following represent signs or symptoms of an unstable patient **EXCEPT**:

1. reduced consciousness level
2. systolic blood pressure <90
3. chest pain
4. nausea and vomiting
5. bilateral rales or other signs of heart failure

11) A 26-year-old man presents with palpitations and is found to be tachycardic. The patient is attached to a monitor, given oxygen and intravenous access is obtained. The monitor shows the following rhythm:

The patient is awake, alert, and denies chest pain or shortness of breath. His BP is 160/92. Which of the following best represents the next step you would take to manage this patient?

1. intravenous beta blocker
2. amiodarone 300mg given intravenously over 20-60 minutes
3. attempt vagal maneuvers
4. synchronized cardioversion at 360 joules (monophasic)
5. defibrillate at 360 joules (monophasic)

12) Which of the following statements is **TRUE** about synchronized cardioversion?

1. a dose of 360 joules (monophasic) or 200 joules (biphasic) is used when you perform synchronized cardioversion for ventricular
2. conscious patients with tachycardias should never be sedated or anesthetized prior to cardioversion
3. the shock must be synchronized with the T wave of the ECG in order to reduce the risk of precipitating ventricular fibrillation
4. As opposed to defibrillation, during cardioversion there is a slight delay between pressing the buttons to shock and the discharge of the shock
5. None of the above statements are true

13) You had resuscitated a patient who is now on a mechanical ventilator. The nurse calls you because his oxygen saturation is falling? All of the following may be performed to evaluate or treat causes of oxygen desaturation **EXCEPT**:

1. Check for displacement of the endotracheal tube
2. Examine the patient (auscultate the lungs, look for tracheal deviation, etc.)
3. Perform in-line suction to remove mucous plugs
4. Remove the patient from the ventilator and manually ventilate the patient with an ambu-bag
5. Assess rectal tone

14) During a resuscitation, you successfully intubate a patient. There is still no return of spontaneous circulation and therefore resuscitation attempts continue. Which of the following is **TRUE** about ventilation after intubation?

1. ambu-bag ventilation should continue at a rate of 30:2 with a pause after compressions to provide ventilations
2. excessive ventilation increases patient survival due to increased intrathoracic pressure
3. ambu-bag ventilation should continue at a rate of approximately 10 ventilations per minute with no pauses in chest compressions to provide ventilations
4. every patient that is intubated during a resuscitation should immediately be connected to a mechanical ventilator

15) You are the SHO at a peripheral hospital when a 65-year-old man presents with new onset right-sided hemiparesis, headache, aphasia and declining mental status. You suspect a cerebrovascular accident (CVA) and decide to intubate the patient due to his low GCS. Now you want to transfer the patient for definitive care. Which of the following actions should you take in order to facilitate a safe transfer?

1. Communicate the clinical history and management with the physician that will receive the patient
2. If available, provide the transfer team with oxygen, monitor, defibrillator, and suction
3. Reassess the patient immediately prior to transfer
4. Secure all tubes, catheters and cannulae
5. All of the above
