# Supplementary material for: The Effectiveness of a ‘Train the Trainer’ Model of Resuscitation Education for Rural Peripheral Hospital Doctors in Sri Lanka
Source: PLoS One. 2013 Nov 8;8(11):e79491. doi: 10.1371/journal.pone.0079491 (PMC3821851; doi:10.1371/journal.pone.0079491)
Supplement: Appendix S4 — Peripheral hospital resuscitation workshop – course content and outline. (DOC) [file pone.0079491.s004.doc]

# Peripheral Hospital training session: course components

| ***Component*** | | ***Topic*** |
| --- | --- | --- |
| **DVD lectures** | | |
|  | Introduction: Resuscitation Training Module overview | |
|  | NEJM (online video) – “How to intubate” | |
|  | ALS Treatment algorithm | |
|  | Tachycardia algorithm & cardioversion | |
|  | Bradycardia algorithm & cardiac pacing | |
| **Skills stations** | | |
|  | Basic Airway management | |
|  | Advanced Airway management | |
| **Scenario Stations** | | |
|  | Initial Resuscitation – PEA/Asystole | |
|  | Resuscitation/Defibrillation – VF/VT | |
|  | Tachycardia/ Bradycardia algorithms - cardioversion & pacing | |
|  | Post resuscitation care | |

# Peripheral Hospital Training Session: course agenda

|  | ***Program for Training Session*** |
| --- | --- |
| **8:00 - 9:30am** | **Pre- assessment (MCQ and Scenarios)** |
| **9:30 - 10:15am** | **Lectures on Airway management** |
| **10:15 - 10:45am** | ***Morning Tea*** |
| **10:45 - 10:45am** | **Practical sessions on Airway management**  **(Skills station 1 and 2)** |
| **11:45 - 12:20pm** | **Lectures on management of cardiac arrest & using the current algorithms for arrhythmias** |
| **12:20 - 13:00pm** | ***Lunch*** |
| **13:00 - 15:00pm** | **Practical sessions:**  **Managing PEA/Asystole (skills station 3)**  **VT/VF/cardiac arrest (skills station 4)**  **Tachycardia and Bradycardia (skills station 5)**  **Post resuscitation care and Transport (Skills station 6)** |
| **15:00 –15:15pm** | ***Afternoon Tea*** |
| **15:15-16:30pm** | **Post – Assessment (MCQ and Scenarios)** |
| **16:30-16:45pm** | **Closing comments**  **(Fill in feedback forms)** |
