# Supplementary material for: The Effectiveness of a ‘Train the Trainer’ Model of Resuscitation Education for Rural Peripheral Hospital Doctors in Sri Lanka
Source: PLoS One. 2013 Nov 8;8(11):e79491. doi: 10.1371/journal.pone.0079491 (PMC3821851; doi:10.1371/journal.pone.0079491)
Supplement: Appendix S5 — Supporting material for trainers & checklists of performance. (DOC) [file pone.0079491.s005.doc]

# Information & Resources for Trainers

The resource manual, the DVDs and the “Instructor Manual” have been given to you to be used as help before during and after the training sessions. Please use the **Instructor Manual** and **Skills Checklist** to guide you during the training sessions.

### Objectives of the Training module

Use the DVD lectures and Skills stations to teach princples outlined in manual:-

- **Recognition** of a critically ill patient – and calling for help
- **Use of a** **system** for assessing a critically ill patient - **ABCDE**
- **Importance of Basic** life saving maneuvers
- **Update of Advanced** **life support** **algorithms** for the arrested patient
  - **The Nov 2005 guidelines (ILCOR)**
- **“Communication” in Resuscitation**
  - Calling for senior support early
  - Communication and leadership during an arrest
  - Post arrest communication with family and other staff
- **Post Resuscitation care & Inter-hospital Transfer**
- **Practical “Hands on” training**
  - Simulation in real life scenarios using CPR mannequins
  - Assessment with constructive feedback

### 4 stage teaching approach for skills stations

Stage 1 – Instructor demonstrates the skill, at normal speed, without explanation

Stage 2 – Instructor demonstrates the skill more slowly, with explanation

Stage 3 – Instructor demonstrates the skill while a participant provides exlanation

Stage 4 – participant demonstrates the skill with explanation

Example of above approach is included in the Trainer’s DVD

### Resuscitation Pack for participants and participant hospitals

1. A4 Colour laminated Algorithm card and Bradycardia/Tachycardia algorithm card (for resuscitation trolley or bag)
2. Wall poster of Advanced life support Algorithm
3. Training DVD

### Checklist for Trainers

### DVD lectures

Lecture topics:

- Introduction
- Airway management
- Intubation
- ALS algorithm
- Tachycardia/ bradycardia
- Post-resuscitation care

### Skills station 1 – Airway management

Airway Opening maneuvers and Adjuncts:

- Head Tilt
- Chin Lift
- Jaw Thrust
- Yankers suction
- Inserts correctly sized Oropharyngeal airway
- Inserts correctly sized Nasopharyngeal airway

Bag mask Ventilation:-

- Assembly of self inflating Ambu bag and mask
- Correct positioning and seal of facemask
- Effective ventilations (causing chest to rise)
- Rate of ventilation 8-10/mins (not >12)
- Ventilating intubated patient (again rate 8-10/mins)
- Single vs. two person technique
- Confirmation of ventilation

###

**Skills station 2 – Advanced airway management**

Preparation and Equipment Check

- Assigns roles
- Ideal equipment and monitoring (PCMALES)
- Check laryngoscope light is working
- Check ET tube – inflates cuff with syringe
- Pre oxygenates (states they would only bag patient if no spontaneous breathing – understands that if patient was breathing themselves bagging may worsen situation)
- Correct positioning (“sniffing the morning air”)

Intubation:

- Suctions first
- Holds laryngoscope in properly (in Left hand)
- If takes longer than 30 seconds – should abort and go back to bagging before second attempt
- Intubates (states whether they saw the cords or not for the team to hear)
- Checks ETT position (breath sounds/ gastric bubbling/ capnography/ etc)
- Instructs person doing Cricoid pressures to “Start cricoid now” (prior to intubating) “remove cricoid” at appropriate times
- Applies Cricoid pressure appropriately (when assisting intubation)

### Skills station 3 - PEA/ Asystole

Initial approach:

- Called for help/ resus team
- Assesses responsiveness
- Uses ABCDE approach
- High flow oxygen
- Initiates CPR when recognizes patient unresponsive

CPR:

- Correct hand position (centre of chest)
- Straight arms
- Compression depth 4-5cm, approx 1/3 of chest
- Allows complete relaxation
- Allows same time for compression as for relaxation
- Starts with chest compressions rather than giving breaths
- Rate 100 per minute
- Ratio 30:2
- Gives 2 breaths after chest compression (each taking 1 second for breath)
- Checks for pulse and rhythm after 2mins of CPR (5 cycles)
- Minimises interruptions to compressions
- If patient comes back to life – states they would put in recovery position

PEA/ asystole:

- Recognition of PEA
- Recognition of asystole
- Recognises that defibrillation is not indicated

### Skills station 4 – VF/VT/Defibrillation

Initial approach:

- Called for help/ resus team
- Assesses responsiveness
- Uses ABCDE approach
- High flow oxygen
- Initiates CPR when recognizes patient unresponsive

CPR:

- Correct hand position (centre of chest)
- Straight arms
- Compression depth 4-5cm, approx 1/3 of chest
- Allows complete relaxation
- Allows same time for compression as for relaxation
- Starts with chest compressions rather than giving breaths
- Rate 100 per minute
- Ratio 30:2
- Gives 2 breaths after chest compression (each taking 1 second for breath)
- Checks for pulse and rhythm after 2mins of CPR (5 cycles)
- Minimises interruptions to compressions
- If patient comes back to life – states they would put in recovery position

Defibrillation

- Recognises pulseless VT/ VF (when CPR stopped)
- Recognises indication for defibrillation and minimizes delay
- Correctly applies paddles/ gel pads/ monitoring
- Selects correct energy level (biphasic/ monophasic)
- Checks and removes hazards (e.g. free-flowing oxygen)
- Warns team to stand clear
- Charges defibrillator, rapid visual check, confirms VT/VF, delivers shock
- Immediately resumes CPR for 2 minutes before rechecking rhythm

### Skills station 5 – Bradycardia / Tachycardia/ Cardioversion

Initial approach:

- Called for help/ resus team
- Assesses responsiveness
- Uses ABCDE approach
- High flow oxygen
- Initiates CPR when recognizes patient unresponsive

Bradycardia:

- Recognition of adverse signs of bradycardia
- Recognition of rhythms that have risks for asystole
- Indication for transcutaneous pacing
- Indication for drugs (e.g. atropine, adrenaline)

Tachycardias

- Recognition of an “unstable” tachycardia
- Indications for, and how to perform synchronized cardioversion
- Distinguishes between broad and narrow complex tachycardias
- Distinguishes between regular and irregular tachycardias
- Understands role of vagal maneuvers and drugs

### Skills station 6 – Post-resuscitation care and transport

Post-resuscitation care:

- Indications for intubation
- Confirms ETT position and secures ETT
- Post-intubation medications
- Supportive care and monitoring (NGT, IDC, cuff pressure, ABG)
- Management of bradycardia, desaturation (DOPE), hypovolemia

Patient transfer:

- Organises transfer with receiving hospital – name, age, sex, condition of patient, reason for transfer, ETA
- Consider need for procedures (e.g. intubation) prior to transfer
- Ongoing assessment and monitoring
- IV fluids
- Oxygen delivery
- Medical staffing plan
- Patient positioning
- Documentation

### Assessments

Assessments

- Pre-course MCQs
- Post-course MCQs
- Post-course scenario
