# Supplementary material for: The Effectiveness of a ‘Train the Trainer’ Model of Resuscitation Education for Rural Peripheral Hospital Doctors in Sri Lanka
Source: PLoS One. 2013 Nov 8;8(11):e79491. doi: 10.1371/journal.pone.0079491 (PMC3821851; doi:10.1371/journal.pone.0079491)
Supplement: Appendix S6 — MCQ test used at assessments. (DOC) [file pone.0079491.s006.doc]

### MCQ Assessment

For all answers choose the single best choice

1. Ten minutes after an 85-year-old woman collapses, paramedics arrive and start CPR for the first time. The monitor shows fine (low-amplitude) VF. Which of the following actions should they take next?

a. Perform at least 5 minutes of vigorous CPR before attempting defibrillation

b. Insert an endotracheal tube, administer 2 to 2.5 mg adrenalin in 10 mL NS through the tube

and then defibrillate

c. Deliver up to 3 precordial thumps while observing the patient’s response on the monitor

d. Deliver about 2 minutes or 5 cycles of CPR, and deliver a 360-J monophasic or equivalent-

current biphasic shock

2. Which of the following facts about identification of Ventricular fibrillation is true?

a. A peripheral pulse that is both weak and irregular indicates VF

b. A sudden drop in blood pressure indicates VF

c. Artifact signals displayed on the monitor can look like VF

d. Turning the signal amplitude (“gain”) to zero can enhance the VF signal

3. A 60-year-old man (weight = 50 kg) with recurrent VF has converted from VF again to a wide- complex non-perfusing rhythm after administration of Adrenalin 1 mg IV and a 3rd shock. Which of the following drug regimens is most appropriate to give next?

a. Amiodarone 300 mg IV push

b. Lidocaine 150 mg IV push

c. Magnesium 3 g IV push, diluted in 10 mL of D5W

d. Procainamide 20 mg/min, up to a maximum dose of 17 mg/kg

4. A cardiac arrest patient arrives in the ED with PEA at 30 bpm. CPR continues, proper tube placement is confirmed, and IV access is established. Which of the following medications is most appropriate to give next?

a. Calcium chloride 5 mL of 10% solution IV

b. Adrenalin 1 mg IV

c. Synchronized cardioversion at 200 J

d. Sodium bicarbonate 1 mEq/kg IV

5. Which of the following causes of PEA is most likely to respond to immediate treatment?

a. Massive pulmonary embolism

b. Hypovolemia

c. Massive acute myocardial infarction

d. Myocardial rupture

6. Which of the following drug-dose combinations is recommended as the initial medication to give a patient in asystole?

a. Adrenalin 3 mg IV

b. Atropine 3 mg IV

c. Adrenalin 1 mg IV

d. Atropine 0.5 mg IV

7. Which of the following causes of out-of-hospital asystole is most likely to respond to treatment?

a. Prolonged cardiac arrest

b. Prolonged submersion in warm water

c. Drug overdose

d. Blunt multisystem trauma

8. Effective bag-mask ventilations are present in a patient in cardiac arrest. Now, 2 minutes after adrenalin 1 mg IV is given, PEA continues at 30 bpm. Which of the following actions should be done next?

a. Administer atropine 3 mg IV

b. Initiate transcutaneous pacing at a rate of 60 bpm

c. Start a dopamine IV infusion at 15 to 20 μg/kg per minute

d. Give adrenalin (1 mL of 1:10 000 solution) IV bolus

9. Which of the following actions helps deliver maximum current during defibrillation?

a. Place alcohol pads between the paddles and skin

b. Reduce the pressure used to push down on the defibrillator paddles

c. Apply conductive paste to the paddles

d. Decrease shock energy after the 2nd shock

10. Which of the following actions is NOT performed when you “clear” a patient just before defibrillator discharge?

a. Check the person managing the airway: body not touching bag mask or tracheal tube, oxygen

not flowing directly onto chest

b. Check yourself: hands correctly placed on paddles, body not touching patient or bed

c. Check monitor leads: leads disconnected to prevent shock damage to monitor

d. Check others: no one touching patient, bed, or equipment connected to patient

11. You prepare to cardiovert an unstable 48-year-old woman with tachycardia. The

monitor/defibrillator is in “synchronization” mode. The patient suddenly becomes

unresponsive and pulseless as the rhythm changes to an irregular, chaotic, VF-like pattern. You charge to 200 J and press the SHOCK button, but the defibrillator fails to deliver a shock. Why?

a. The defibrillator/monitor battery failed

b. The “sync” switch failed

c. You cannot shock VF in “sync” mode

d. A monitor lead has lost contact, producing the “pseudo-VF” rhythm

12. A woman with a history of narrow-complex PSVT arrives in the ED. She is alert and oriented but pale. HR is 165 bpm, and the ECG documents SVT. BP is 105/70 mm Hg. Supplemental oxygen is provided, and IV access has been established. Which of the following drug-dose combinations is the most appropriate initial treatment?

a. Adenosine 6 mg rapid IV push

b. Adrenalin 1 mg IV push

c. Synchronized cardioversion with 25 to 50 J

d. Atropine 1 mg IV push

13. A 34-year-old woman with a history of mitral valve prolapse presents to the ED complaining of palpitations. Her vital signs are as follows: HR = 165 bpm, resp = 14 per minute, BP = 118/92 mm Hg, and O2 sat = 98%. Her lungs sound clear, and she reports no shortness of breath or dyspnea on exertion. The ECG and monitor display a narrow-complex, regular tachycardia. Which of the following terms best describes her condition?

a. Stable tachycardia

b. Unstable tachycardia

c. Heart rate appropriate for clinical condition

d. Tachycardia secondary to poor cardiovascular function

14. A 75-year-old man presents to the ED with a 1-week history of lightheadedness, palpitations, and mild exercise intolerance. The initial 12-lead ECG displays atrial fibrillation, which continues to show on the monitor at an irregular HR of 120 to 150 bpm and a BP of 70/40mm Hg. Which of the following therapies is the most appropriate next intervention?

a. Call a cardiologist for assistance

b. Lidocaine 1 to 1.5mg/kg IV bolus

c. Amiodarone 300 mg IV bolus

d. Immediate cardioversion

15. A 25-year-old woman presents to the ED and says she is having another episode of PSVT. Her medical history includes an electrophysiologic stimulation study (EPS) that confirmed a reentry tachycardia, no Wolff-Parkinson-White syndrome, and no preexcitation. HR is 180 bpm. The patient reports palpitations and mild shortness of breath. Vagal maneuvers with carotid sinus massage have no effect on HR or rhythm. Which of the following is the most appropriate next intervention?

a. DC cardioversion

b. IV diltiazem

c. IV propranolol

d. IV adenosine

16. Endotracheal intubation has just been attempted for a patient in respiratory arrest. During bag-mask ventilation you hear stomach gurgling over the epigastrium but no breath sounds, and oxygen saturation (per pulse oximetry) stays very low. Which of the following is the most likely explanation for these findings?

a. Intubation of the esophagus

b. Intubation of the left main bronchus

c. Intubation of the right main bronchus

d. Bilateral tension pneumothorax

17. Which of the following statements correctly describes the ventilations that should be provided after endotracheal tube insertion, cuff inflation, and verification of tube position?

a. Deliver 8 to 10 ventilations per minute with no pauses for chest compressions

b. Deliver ventilations as rapidly as possible as long as visible chest rise occurs with each breath

c. Deliver ventilations with a tidal volume of 3 to 5 mL/kg

d. Deliver ventilations using room air until COPD is ruled out

18. When intubating during an arrest which of the following is INCORRECT

a. Only intubate if you have been properly trained and have adequate experience

b. Stop chest compressions while intubation is being carried out

c. Pre-oxygenation with high concentration oxygen should always be performed when possible

d. After the patient is intubated the ratio of chest compression to ventilations is 30:2

19. Regarding bag-mask ventilation which of the following is correct?

a. Ambu bags are universal in size so that the same one can be used effectively for all patients

b. The rate of ventilation should be approximately 10 ventilations per min in an arrest situation, but this rate should be increased if the patient is hypoxic

c. Effective ventilation cannot be achieved unless there is a good seal between the face mask and the patient’s face

d. When oxygenating a patient prior to intubation one should actively ventilate the patient, even if the patient is breathing himself

e. If you are performing bag mask ventilation, you do not need to have an airway adjunct such as an oropharangeal airway in place

20. Which of these statements about IV administration of medications during attempted resuscitation is true?

a. Give adrenalin via the intracardiac route if IV access is not obtained within 3 minutes

b. Follow IV medications through peripheral veins with a fluid bolus

c. Do not follow IV medications through central veins with a fluid bolus

d. Run normal saline mixed with sodium bicarbonate (100 mEq/L) during continuing CPR

21. A patient with a heart rate of 40 bpm is complaining of chest pain and is confused. After oxygen, what is the first drug you should administer to this patient while a transcutaneous pacer is brought to the room?

a. Atropine 0.5 mg

b. Adrenalin 1 mg IV push

c. Isoproterenol infusion 2 to 10 μg/min

d. Adenosine 6 mg rapid IV push

22. Which of the following rhythms is a proper indication for transcutaneous cardiac pacing?

a. Sinus bradycardia with no symptoms

b. First degree AV block

c. Complete heart block with pulmonary edema

d. Asystole that follows 6 or more defibrillation shocks

23. A patient with an HR of 30 to 40 bpm complains of dizziness, cool and clammy extremities, and dyspnea. He is in third-degree AV block. All treatment modalities are present. What would you do first?

a. give atropine 1 mg IV

b. give adrenalin 1 mg IV push

c. start dopamine infusion 2 to 10 μg/min

d. begin immediate transcutaneous pacing, sedate if possible

24. In a conscious patient all of the following are signs of a patient being critically ill EXCEPT for:

a. A sudden fall in GCS < 2

b. HR <40 or >140

c. RR <5 RR>36

d. Fever of > T 38.5

e. Systolic BP <90

25. Regarding “Circulation” in an arrest situation which is correct?

a. Patients are unlikely to need IV fluids in an arrest

b. CPR and fluid resuscitation are unlikely to save the life of someone with Ventricular Fibrillation until a DC shock is given

c. The best place to put an IV cannula in arrest is over the radial side of the wrist

d. Even if a no central pulses are felt, it is still useful to check blood pressure during resuscitation of a cardiac arrest

e. After ROSC (return of spontaneous circulation) with a pulse felt peripherally, there is never any reason to check the blood pressure

Rhythm Recognition

Identify the Following Rhythms (write the single best choice)

26.

a. Normal Sinus Rhythm

b. Sinus Tachycardia

c. Sinus Bradycardia

d. Atrial Fibrillation

e. Atrial Flutter

f. Reentry Supraventricular Tachycardia

g. Monomorphic Ventricular Tachycardia

h. Polymorphic Ventricular Tachycardia

i. Ventricular Fibrillation

j. Second-Degree Atrioventricular Block

h. Third-Degree Atrioventricular Block

27.

a. Normal Sinus Rhythm

b. Sinus Tachycardia

c. Sinus Bradycardia

d. Atrial Fibrillation

e. Atrial Flutter

f. Reentry Supraventricular Tachycardia

g. Monomorphic Ventricular Tachycardia

h. Polymorphic Ventricular Tachycardia

i. Ventricular Fibrillation

j. Second-Degree Atrioventricular Block

h. Third-Degree Atrioventricular Block

28.

a. Normal Sinus Rhythm

b. Sinus Tachycardia

c. Sinus Bradycardia

d. Atrial Fibrillation

e. Atrial Flutter

f. Reentry Supraventricular Tachycardia

g. Monomorphic Ventricular Tachycardia

h. Polymorphic Ventricular Tachycardia

i. Ventricular Fibrillation

j. Second-Degree Atrioventricular Block

h. Third-Degree Atrioventricular Block

29.

a. Normal Sinus Rhythm

b. Sinus Tachycardia

c. Sinus Bradycardia

d. Atrial Fibrillation

e. Atrial Flutter

f. Reentry Supraventricular Tachycardia

g. Monomorphic Ventricular Tachycardia

h. Polymorphic Ventricular Tachycardia

i. Ventricular Fibrillation

j. Second-Degree Atrioventricular Block

h. Third-Degree Atrioventricular Block

30.

a. Normal Sinus Rhythm

b. Sinus Tachycardia

c. Sinus Bradycardia

d. Atrial Fibrillation

e. Atrial Flutter

f. Reentry Supraventricular Tachycardia

g. Monomorphic Ventricular Tachycardia

h. Polymorphic Ventricular Tachycardia

i. Ventricular Fibrillation

j. Second-Degree Atrioventricular Block

h. Third-Degree Atrioventricular Block

Answers

1. d

2. c

3. a

4. b

5. b

6. c

7. c

8. a

9. c

10. c

11. c

12. a

13. a

14. d

15. d

16. a

17. a

18. d

19. c

20. b

21. a

22. c

23. d

24. d

25. b

26. g

27. h

28. e

29. i

30. j
