# Supplementary material for: The Effectiveness of a ‘Train the Trainer’ Model of Resuscitation Education for Rural Peripheral Hospital Doctors in Sri Lanka
Source: PLoS One. 2013 Nov 8;8(11):e79491. doi: 10.1371/journal.pone.0079491 (PMC3821851; doi:10.1371/journal.pone.0079491)
Supplement: Appendix S7 — Script for resuscitation scenario & picture of assessment room. (DOC) [file pone.0079491.s007.doc]

# Scenario Assessment script & instructions

### Scenario

“When you go behind the screen you will see a mannequin in a hospital bed– we want you to imagine this is a 50 year old man who has collapsed and is unresponsive. You see this patient when you are walking to your clinic.

When I say start I want you to do as you would in real life. During the scenario I will answer any questions you have about the patient’s condition, which you cannot work out for yourself and that you “look for”. But I do not want you to talk to me - You are alone. The only equipment you have is what you see around you.”

*[read scenario twice. Press record on the video camera]*

“Just one moment as I announce your ID for the video”

*[say PHP No…. Scenario 1]*

“Do what you would do in real life – continue doing so until I tell you to stop. Start NOW”

*[start stopwatch, play on computer also]*

***End of Scenario***

Assessor steps in and says “And help arrives!!”

Evaluator instructions (scripted responses):

| ***Action*** | ***Response*** |
| --- | --- |
| Checks for pulse | Say “there is no pulse” |
| Checks for breathing | There is no chest movement |
| If participant starts to give expired air ventilation ***or*** attempts to give expired air ventilation | “if you want to give ventilations, please use the Ambu bag provided” |
| If patient asks for details of the patient, eg “what is the patient’s name” or “how old is he” | “You do not know his name, he is a 50 year old male who has collapsed” |
| I would like a “………” eg Monitor | The only equipment you have immediately available is what you see around you. You are alone. Do as you would in real life |
| I would like some help | Help is on its way |

After **1 min** of the participant performing single rescuer CPR the evaluator stops them and thanks them – immediately go on to scenario 2.

### Time prompts

| ***Time*** | ***Prompt*** |
| --- | --- |
| IF no CPR started by 1 min | “I want you to start cardiopulmonary resuscitation on this patient please” |
| After 1 min of initiation of compressions | Say “And help arrives”. Please come out of the room” [ie. step behind the screen]” |
|  |  |

Setting:

RA Skill trainer Mannequin in standard hospital bed.

No pulse, No breathing.

Ambu bag and mask is on the bedside table next to patient (not on bed)

Screen covering mannequin from starting point of participant – all scenario briefing to be given out of the line of vision of mannequin, behind the screen.

Assessor stands on the ***other side*** of the screen to the participant (out of sight but within earshot) – Assessor makes limited scripted responses as appropriate.

***Figure*** *. Example of a peripheral hospital Assessment station setup*

###
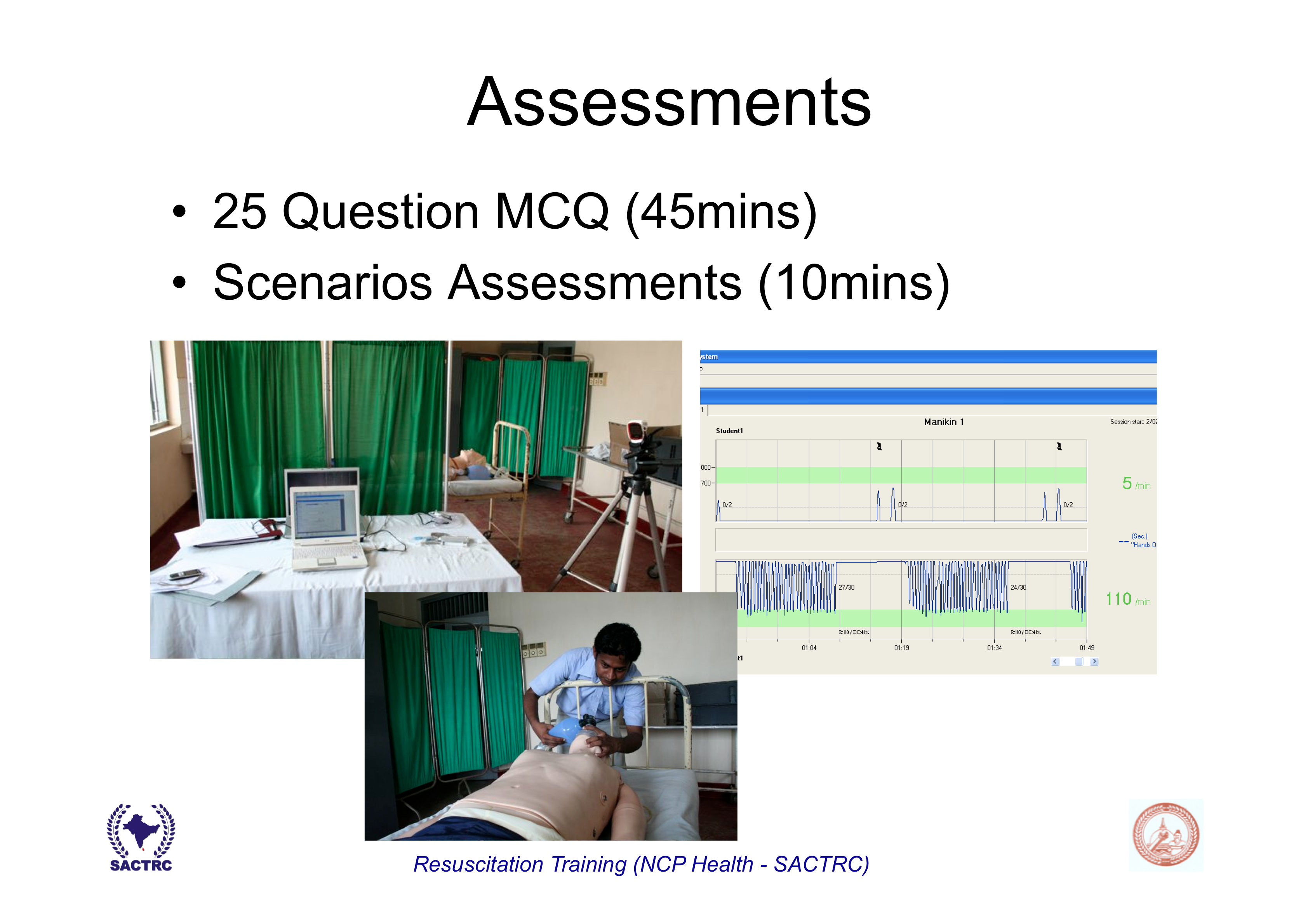


# Equipment

##### Essential

1. CPR recording Mannequin (see below)
2. Hospital Bed & Mattress x1
3. Ambu bags x2
4. Laryngoscope and x2 blades (Mac 3 and Mac4
5. ET tube with introducer
6. Oxygen mask and tubing
7. Poster with ALS algorithm x2

##### Non essential (but valuable for simulation )

1. Drip stand
2. Hospital bedside table
3. Old saturation monitor
4. Old defibrillation unit (Better to have a functional defibrillator but this might be economically challenging)

**Mannequin requirements**

- Full body (for realism)
- Continuous real time feed back for ventilation and compression (LED lights)
- Skill reporting (ability to generate a print-out of the performance, or store this data on a computer.)
- A head piece that allows airway opening manoeuvres and INTUBATION
- A body that can simulate arrhythmias and defibrillation. A computer scenario program that comes with the mannequin so that data can be **recorded** an **stored** on a computer
