# Supplementary figures and images for: The Effectiveness of a ‘Train the Trainer’ Model of Resuscitation Education for Rural Peripheral Hospital Doctors in Sri Lanka
Source: PLoS One. 2013 Nov 8;8(11):e79491. doi: 10.1371/journal.pone.0079491 (PMC3821851; doi:10.1371/journal.pone.0079491)

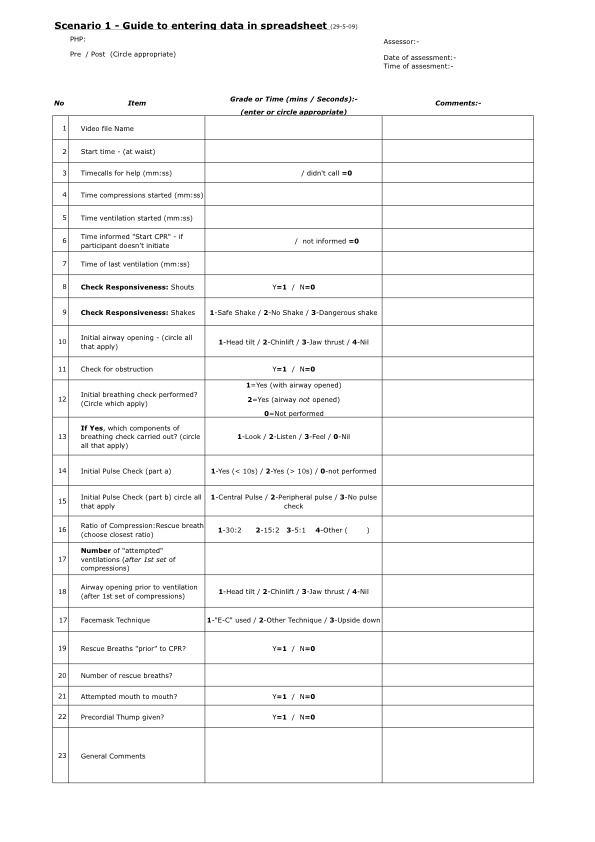

Supplement: Appendix S8 — Marking schedule for video assessment. (DOCX) [file pone.0079491.s008.docx]
